# Resistance to Selective FGFR Inhibitors in *FGFR-*Driven Urothelial Cancer

**DOI:** 10.1158/2159-8290.CD-22-1441

**Published:** 2023-06-28

**Authors:** Francesco Facchinetti, Antoine Hollebecque, Floriane Braye, Damien Vasseur, Yoann Pradat, Rastislav Bahleda, Cédric Pobel, Ludovic Bigot, Olivier Déas, Juan David Florez Arango, Giorgia Guaitoli, Hayato Mizuta, David Combarel, Lambros Tselikas, Stefan Michiels, Sergey I. Nikolaev, Jean-Yves Scoazec, Santiago Ponce-Aix, Benjamin Besse, Ken A. Olaussen, Yohann Loriot, Luc Friboulet

**Affiliations:** 1 Université Paris-Saclay, Gustave Roussy, Inserm U981, Villejuif, France.; 2 Département d'Innovation Thérapeutique (DITEP), Gustave Roussy, Villejuif, France.; 3 Département de Médecine Oncologique, Gustave Roussy, Villejuif, France.; 4 Medical Biology and Pathology Department, Gustave Roussy, Villejuif, France.; 5 AMMICa UAR3655/US23, Gustave Roussy, Villejuif, France.; 6 Université Paris-Saclay, CentraleSupélec, MICS Lab, Gif-Sur-Yvette, France.; 7 XenTech, Evry, France.; 8 PhD Program Clinical and Experimental Medicine, University of Modena and Reggio Emilia, Modena, Italy.; 9 BIOTHERIS, Department of Interventional Radiology, Gustave Roussy, Université Paris-Saclay, Villejuif, France.; 10 Université Paris-Saclay, Inserm, CESP, Villejuif, France.; 11 Gustave Roussy, Office of Biostatistics and Epidemiology, Villejuif, France.; 12 Université Paris-Saclay, Faculté de Médecine, Le Kremlin Bicêtre, France.

## Abstract

Several fibroblast growth factor receptor (FGFR) inhibitors are approved or in clinical development for the treatment of *FGFR*-driven urothelial cancer, and molecular mechanisms of resistance leading to patient relapses have not been fully explored. We identified 21 patients with *FGFR*-driven urothelial cancer treated with selective FGFR inhibitors and analyzed postprogression tissue and/or circulating tumor DNA (ctDNA). We detected single mutations in the FGFR tyrosine kinase domain in seven (33%) patients (FGFR3 N540K, V553L/M, V555L/M, E587Q; FGFR2 L551F) and multiple mutations in one (5%) case (FGFR3 N540K, V555L, and L608V). Using Ba/F3 cells, we defined their spectrum of resistance/sensitivity to multiple selective FGFR inhibitors. Eleven (52%) patients harbored alterations in the PI3K–mTOR pathway (*n* = 4 *TSC1/2*, *n* = 4 *PIK3CA*, *n* = 1 *TSC1* and *PIK3CA*, *n* = 1 *NF2*, *n* = 1 *PTEN*). In patient-derived models, erdafitinib was synergistic with pictilisib in the presence of PIK3CA E545K, whereas erdafitinib–gefitinib combination was able to overcome bypass resistance mediated by EGFR activation.

**Significance:**

In the largest study on the topic thus far, we detected a high frequency of FGFR kinase domain mutations responsible for resistance to FGFR inhibitors in urothelial cancer. Off-target resistance mechanisms involved primarily the PI3K–mTOR pathway. Our findings provide preclinical evidence sustaining combinatorial treatment strategies to overcome bypass resistance.

*See related commentary by Tripathi et al., p. 1964.*

*This article is featured in Selected Articles from This Issue, p. 1949*

## Teaser

In patients with *FGFR*-driven urothelial cancer, resistance to selective FGFR inhibitors is largely explained by the emergence of *FGFR* kinase domain mutations and alterations in the PI3K–mTOR pathway.

## INTRODUCTION

Molecular alterations in the fibroblast growth factor receptor (*FGFR*) gene family are frequent events in urothelial cancer. *FGFR3* mutations, a hallmark of low-grade, non–muscle invasive bladder cancer, are present in 12% to 22% of high-grade, muscle-invasive tumors (1–4). Upper tract urothelial carcinomas, representing less than 10% of urothelial cancers, harbor *FGFR3* mutations in up to 60% of high-grade cases (1, 4, 5). FGFR3 activating mutations occur mainly in the extracellular domain of FGFR3 (S249C, R248C, and Y373C hotspots; refs. 3–5). Oncogenic *FGFR3* fusions (with *TACC3* as the most frequent partner gene) are found in up to 2% to 3% of bladder cancer and detected in upper tract urothelial carcinoma as well (2, 4–6). Activating alterations in other *FGFR* genes, although rare, are also reported in urothelial tumors (7, 8).

Oncogenic activating *FGFR* alterations in urothelial carcino­mas represent an opportunity for targeted therapeutic intervention with selective FGFR inhibitors. In a phase II trial leading to FDA approval of erdafitinib, 101 patients with *FGFR3* mutations or *FGFR2/3* rearrangements treated with erdafitinib at the starting dose of 8 mg daily, the objective response rate (ORR) and median progression-free survival (PFS) were 40% and 5.5 months, respectively (9, 10). Two additional reversible inhibitors, infigratinib and pemigatinib, and the irreversible inhibitor futibatinib led to slightly inferior outcomes in FGFR-selected populations of patients with urothelial cancer; however, the futibatinib data were obtained mainly from a population pretreated with FGFR inhibitors (11–13). Recently, in a phase II trial, the reversible inhibitor rogaratinib did not show superiority to chemotherapy in pretreated patients selected for FGFR1/3 mRNA overexpression. Among the 21 patients with *FGFR3* mutations or fusions randomized to receive rogaratinib, responses were observed in 11 cases (ORR 52.4%); four out of 15 patients with FGFR3 molecular alterations treated with chemotherapy achieved disease response (ORR 26.7%; ref. 14).

In line with other oncogene-driven diseases, both primary and acquired resistance limit the efficacy of FGFR inhibitors in urothelial carcinomas. Evidence regarding mechanisms of resistance to FGFR inhibitors is limited to the analysis of 22 circulating tumor DNA (ctDNA) samples at progression on infigratinib. In four patients, acquired mutations in the FGFR3 tyrosine kinase domain were detected (FGFR3 V555L, V555M, and L608V, according to the NM_000142.4 transcript; ref. 11).

Here we present a molecular and functional study of resistance to selective FGFR inhibitors in patients with *FGFR*-driven urothelial cancer. FGFR tyrosine kinase domain mutations and off-target molecular activations were frequently observed. According to the resistance mechanisms recorded, we evaluated adaptive (i.e., alternative FGFR inhibitors) and combinatorial treatment strategies in preclinical models, providing potential future guidance for clinical application.

## RESULTS

### Molecular Landscape of Advanced/Metastatic Urothelial Cancers

We collected clinical and molecular data for a total of 56 patients with advanced/metastatic urothelial cancer (*n* = 39 bladder, *n* = 15 upper urothelial tract, *n* = 2 with both localizations). Thirty-eight patients had both whole-exome sequencing (WES) and RNA sequencing (RNA-seq) performed on tissue biopsy, nine had WES only, and one had RNA-seq only; eight patients had a targeted next-generation sequencing (NGS) panel on ctDNA and/or tissue biopsy.

**Figure 1.**
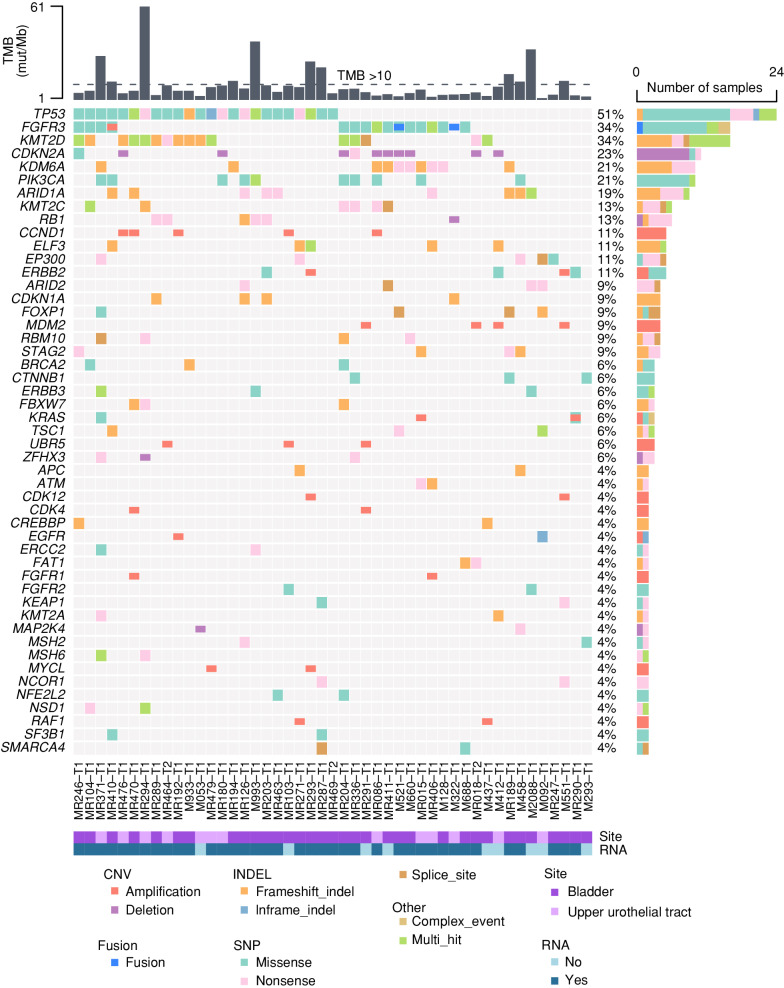
Mutational landscape of urothelial cancers included in MATCH-R or MOSCATO studies, with WES ± RNA-seq performed. CNV, copy-number variation; INDEL, insertion/deletion; mut/Mb, mutations per megabase; SNP, single-nucleotide polymorphism; TMB, tumor mutation burden.

The assessment of the mutational features has been the main objective of seminal studies shedding light on the frequency of *FGFR3* alterations and other molecular features in urothelial carcinomas, focusing on localized diseases (2, 4, 5). We therefore performed a global mutational landscape analysis using tumor samples from the 47 urothelial cancer patients with advanced/metastatic disease and WES available (Fig. 1). The most frequently altered genes were *TP53* (51%), *FGFR3* (34%), and *KMT2D* (34%). Taking into account the limitation of evaluating expression profiles from bulk RNA-seq analyses of tissue biopsies with variable tumor/stroma cellularity, we investigated differential expression of genes involved in the main signaling pathways in the 39 samples with RNA-seq available. No clear pattern was highlighted between *FGFR3*-driven and non–*FGFR3*-driven patient samples with the exception of *FGFR3* mRNA (Supplementary Fig. S1A). Analyzing FGFR family member expression levels specifically, we did indeed reveal a strong significant overexpression of *FGFR3* mRNA in *FGFR3*-driven samples compared with *FGFR3* wild-type (WT) ones (Supplementary Fig. S1B). By contrast, only a slight decrease was observed for *FGFR1* and *FGFR4* mRNA and no difference was observed for *FGFR2* expression.

### Patients Progressing on Selective FGFR Inhibitors

**Figure 2.**
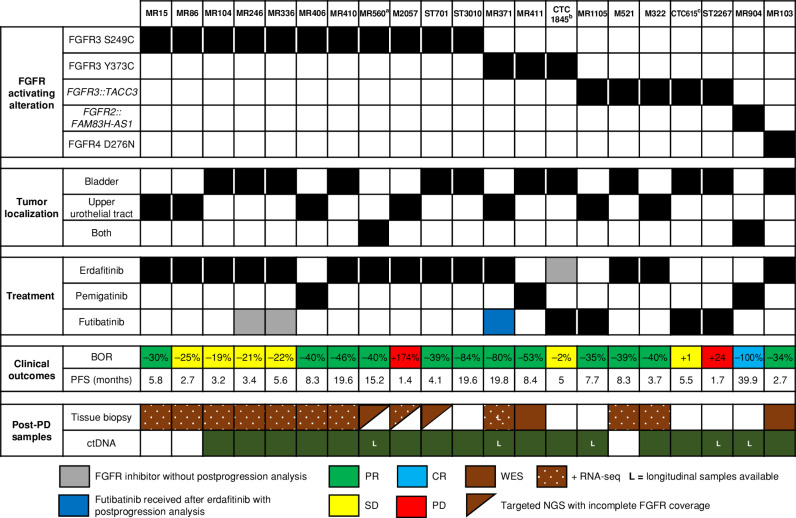
Overview of patients with urothelial cancer progressing on FGFR inhibitors, treatment outcomes, and postprogression sample availability. Granular view at the individual patient level. BOR, best objective response; CR, complete response; PD, progressive disease; PR, partial response; SD, stable disease. ^a^Patient MR560 received erdafitinib combined with a PD-1 inhibitor. ^b^Patient CTC1845 was treated with a sequence of erdafitinib–futibatinib (with intervening immunotherapy between the two FGFR inhibitors), and the ctDNA sample was collected at progression on futibatinib. ^c^Patient CTC615 received pazopanib before futibatinib.

We then focused on the 21 patients treated with selective FGFR inhibitors; 12 and seven had bladder and upper tract tumors, respectively, and in two additional cases both localizations were present. Eleven cases harbored an FGFR3 S249C mutation, five had an *FGFR3*::*TACC3* fusion, and three displayed an FGFR3 Y373C mutation. One patient (MR904) harbored an *FGFR2::FAM83H-AS1* rearrangement and one (MR103) an FGFR4 D276N mutation (Fig. 2; Supplementary Table S1).

Postprogression samples were collected on erdafitinib (*n* = 14), futibatinib (*n* = 4), or pemigatinib (*n* = 3). As best objective responses, we observed one patient with a complete response (CR), 12 patients with partial responses (PR), six patients with stable disease (SD), and two patients with progressive disease (PD). Of note, all patients were previously treated for advanced disease, and the majority received the FGFR inhibitor as the second treatment line (range, second–sixth), but no clear correlation between the type of cytotoxic agents received by patients prior to FGFR inhibitors and molecular alterations could be evidenced (Supplementary Table S2).

Twelve patients had both postprogression tissue biopsy and ctDNA, six had ctDNA only, and three had tissue only. Four patients received futibatinib after having progressed to either erdafitinib or pemigatinib. Longitudinal analysis was possible for 5 patients thanks to additional ctDNA samples, including one patient with tissue molecular profiles obtained both at erdafitinib and futibatinib progression. Overall, we analyzed 39 postprogression samples: 16 postprogression tissue analysis and 23 ctDNA samples.

### Molecular Findings at Progression on Selective FGFR Inhibitors in Urothelial Cancer

#### FGFR Kinase Domain Mutations

**Figure 3.**
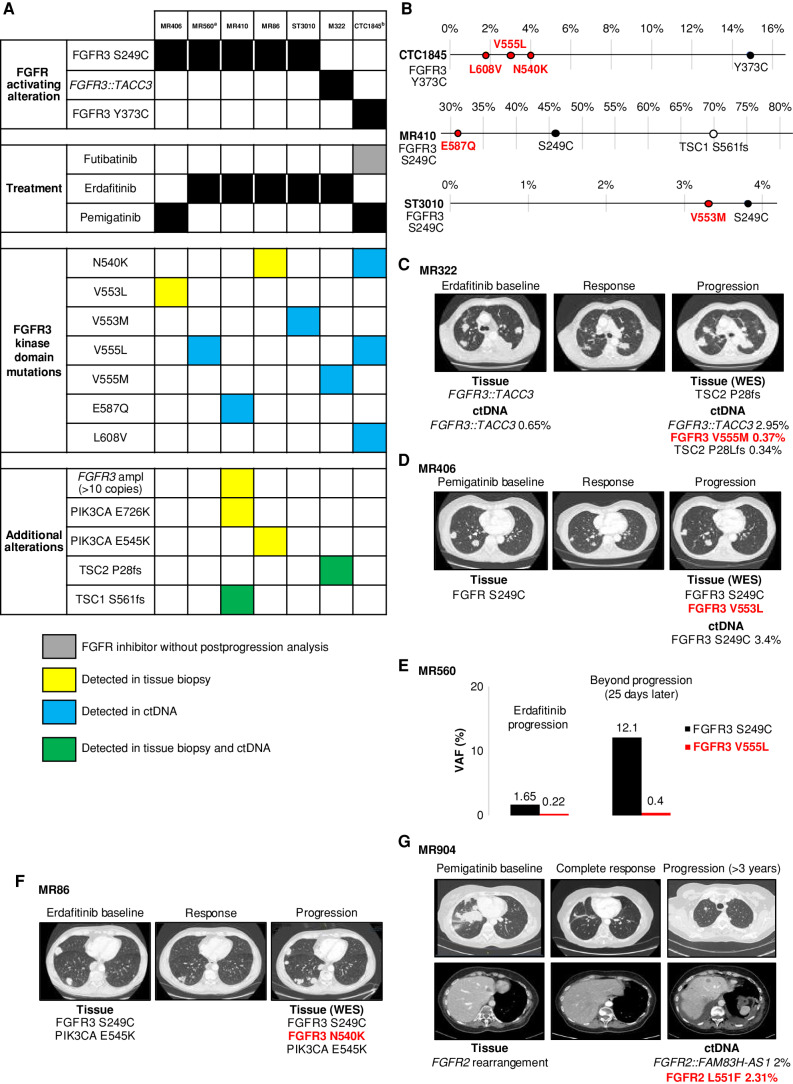
FGFR kinase domain mutations detected at progression on FGFR inhibitors in urothelial cancer. **A,** Heat map representing individual patient data among the cohort of *FGFR3*-driven urothelial cancers. **B**–**G,** Molecular and radiologic findings. Mutations and fusions in ctDNA are reported as variant allele frequencies (VAF). Mutations in the FGFR tyrosine kinase domain are reported in red. ampl, amplification. ^a^Patient MR560 received erdafitinib combined with a PD-1 inhibitor. ^b^Patient CTC1845 was treated with a sequence of erdafitinib–futibatinib (with intervening immunotherapy between the two FGFR inhibitors) before the ctDNA sample was collected at progression on futibatinib.

In the cohort of 19 *FGFR3*-driven urothelial cancers that progressed on erdafitinib, pemigatinib, or futibatinib, FGFR3 kinase domain mutations were detected in seven patients (37%; Fig. 3A). Sequencing data of pretreatment tissue biopsy and/or ctDNA were available for five out of seven patients (71%), and no preexisting FGFR3 kinase domain mutation was detected in those baseline samples. Of note, the activating *FGFR3* alterations were detected in all ctDNA and tissue samples available at progression. In six patients, we detected single mutations in the FGFR3 kinase domain in either ctDNA or tissue biopsy: N540K, V553L, V553M, V555L, V555M, and E587Q (Fig. 3A). Three concomitant FGFR3 kinase domain mutations (N540K, V555L, and L608V), in addition to the activating Y373C, were identified in the ctDNA of patient CTC1845 (Fig. 3A and B), who progressed on futibatinib after having received erdafitinib (PR, 67%; PFS, 8.6 months). The allelic status (*cis*/*trans* configuration) of these four FGFR3 mutations could not be assessed due to ctDNA amplicon size limitation.

Patient MR410 had an FGFR3 E587Q mutation detected in ctDNA only at progression on erdafitinib, a TSC1 S561fs alteration detected both in tissue and in liquid biopsies, whereas *FGFR3* amplification and PIK3CA E726K mutation were found in the tissue biopsy only (Fig. 3B).

In the erdafitinib “baseline” ctDNA sample of patient M322, we observed an *FGFR3::TACC3* fusion, later detected at higher variant allele frequency (VAF) in the postprogression liquid biopsy, together with an FGFR3 V555M mutation and a *TSC2* frameshift event (P28Lfs; Fig. 3C).

In line with limited ctDNA shedding in cases of intrathoracic-only disease, the FGFR3 V553L mutation was detected only in the tissue biopsy and not in the ctDNA of patient MR406, who experienced mild, thoracic-only progression on pemigatinib (Fig. 3D). Given the availability of mRNA from the tissue biopsy, we performed TOPO-TA cloning and observed FGFR3 S249C and V553L mutations on the same allele, confirming that the mutational events arose in *cis* with the driver mutation (Supplementary Fig. S2).

The VAF increase of both FGFR3 S249C and V555L reflected the progression during erdafitinib treatment in patient MR560 (Fig. 3E).

For patient MR86, an FGFR3 N540K mutation was detected in tissue biopsy in addition to the driver mutation FGFR3 S249C and a PIK3CA E545K mutation already existing in the pre-erdafitinib sample (Fig. 3F).

Additionally, upon detection of an *FGFR2* rearrangement in one patient with urothelial cancer (MR904), third-line pemigatinib resulted in a CR (Fig. 3G). Upon progression (after 3 years and 4 months), ctDNA analysis revealed the fusion partner (*FGFR2::FAM83H-AS1*), as well as an FGFR2 L551F mutation (according to NM _022970.3 transcript).

#### Molecular Alterations in the PI3K–mTOR Pathway

In 11 of 19 patients (58%) with *FGFR3-*driven urothelial carcinoma, we detected an alteration in the PI3K–mTOR pathway in postprogression and/or in baseline samples (Table 1).

**Table 1.** Off-target molecular alterations in the PI3K–mTOR pathway detected in patients suffering from *FGFR3*-driven urothelial cancers

Patient	Baseline tissue analysis	Baseline ctDNA (VAF)	FGFR inhibitor	BOR	PFS (months)	Postprogression tissue analysis	Postprogression ctDNA analysis (VAF)
**MR15**	FGFR3 S249C	NA	Erdafitinib	−30%	5.8	FGFR3 S249C	NA
	PIK3CA E545A					PIK3CA E545A	
**MR86**	FGFR3 S249C	NA	Erdafitinib	−25%	2.7	FGFR3 S249C	NA
	PIK3CA E545K					**FGFR3 N540K**	
						PIK3CA E545K	
**ST2267**	*FGFR3::TACC3*	*FGFR3::TACC3* 8.03%	Futibatinib	+24%	1.7	NA	*FGFR3::TACC3* 4.16%
	PIK3CA H1047R						
**MR336**	FGFR3 S249C	FGFR3 S249C 7.72%	Erdafitinib	−22%	5.6	FGFR3 S249C	FGFR3 S249C 10.38%
						**PIK3CA E545K**	**PIK3CA E545K 7%**
**MR410**	FGFR3 S249C	NA	Erdafitinib	−46%	19.6	FGFR3 S249C	FGFR3 S249C 45.94%
	TSC1 S561fs					*FGFR3* ampl (>10 copies)	**FGFR3 E589Q 31.12%**
						TSC1 S561fs	TSC1 S561fs 69.96%
						**PIK3CA E726K**	
**M322**	*FGFR3::TACC3*	*FGFR3::TACC3* 0.65%	Erdafitinib	−40%	3.7	**TSC2 P28fs**	*FGFR3:TACC3* 2.95%
							**FGFR3 V555M 0.37%**
							**TSC2 P28fs 0.34%**
**M521**	*FGFR3::TACC3*	NA	Erdafitinib	−39%	8.3	*FGFR3::TACC3*	NA
						TSC1 Q865*	
**MR1105**	*FGFR3::TACC3*	NA	Futibatinib	−35%	7.7	NA	*FGFR3::TACC3* 9.8%
							**TSC1 A186fs 1.8%**
**ST701**	NA	FGFR3 S249C 0.2%	Erdafitinib	−39%	4.1	TSC1 Q830*	FGFR3 S249C 0.45%
							TSC1 Q830* 1.8%
**MR246**	FGFR3 S249C	NA	Erdafitinib	−21%	3.4	FGFR3 S249C	FGFR3 S249C 2.37%
							**NF2 L163fs 0.21%**
							**FGFR2 V517M 0.12%**
**M2057**	FGFR3 S249C	NA	Erdafitinib	+174%	1.4	**PTEN C136fs**	FGFR3 S249C 48.13%
						**FGFR3 CNV (6 copies)**	

NOTE: Eleven out of 19 (58%) patients harbored alterations in genes belonging to the PI3K–mTOR pathway, with an enrichment in postprogression samples. Molecular alterations in bold were not present in baseline samples.

Abbreviations: ampl, amplification; BOR, best objective response; CNV, copy-number variation; NA, not assessed.

In five samples, a *PIK3CA* activating mutation was detected at progression, but in three cases the *PIK3CA* alteration was present before the treatment with FGFR inhibitors (patients MR15, MR86, and ST2267). Patients MR15 and MR86 achieved clinical responses on erdafitinib before progression. Patient MR86 acquired an FGFR3 N540K mutation, whereas an EGFR hyperphosphorylation [determined by a phospho–receptor tyrosine kinase (RTK) array] potentially explained the resistance for patient MR15 (see the section “Nonmutational Resistance Mechanism Identified Using Patient-Derived Models”). By contrast, patient ST2267, who had a baseline PIK3CA H1047R tumor mutation, experienced primary resistance to futibatinib. For patient MR336, the PIK3CA E545K mutation was acquired during treatment with erdafitinib, which potentially conferred the primary resistance to futibatinib (Supplementary Fig. S3A).

In addition to *PIK3CA* mutations, *TSC1/2* inactivating mutations were also frequently detected (5/19) in patients progressing on FGFR inhibitors (Table 1). Patient MR410 had a TSC1 S561fs mutation detected in both postprogression (erdafitinib) tissue and ctDNA (VAF 69.96%), concurrent with PIK3CA E726K and FGFR3 E589Q (VAF 31.12%) mutations.

A TSC2 P28Lfs mutation in patient M322, a TSC1 Q865* mutation in patient M521, and a TSC1 Q830* mutation in patient ST701 emerged at progression on erdafitinib, and a TSC1 A186fs mutation in patient MR1105 emerged at futibatinib progression (Supplementary Fig. S3B).

In patient MR246, who had an FGFR3 S249C-driven bladder cancer that ultimately progressed on erdafitinib, an NF2 frame­shift event was detected by ctDNA together with an FGFR2 V517M mutation.

Finally, a PTEN C136fs mutation was detected in a patient with primary resistance to erdafitinib (M2057).

The frequency of these mutations arising in tumor suppressor genes suggests they should be considered as potential targets for overcoming treatment resistance.

### Functional Analyses of Molecular Alterations and Overcoming Therapeutic Strategies

#### FGFR Kinase Domain Mutations Confer Resistance to Selective FGFR Inhibitors

**Figure 4.**
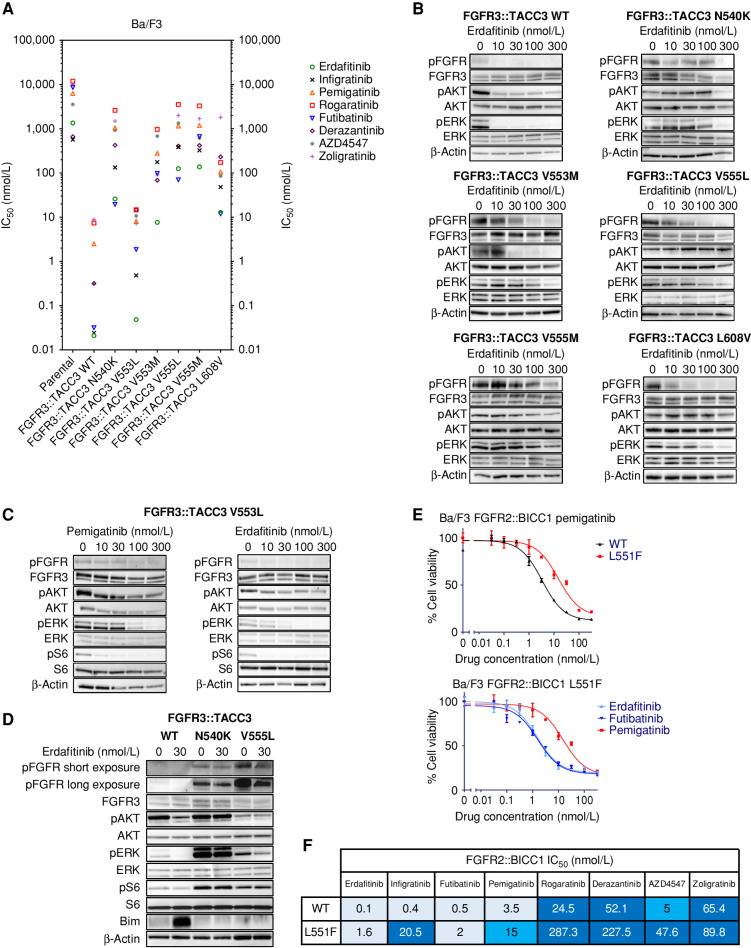
Functional characterization of FGFR2/3 kinase domain mutations in Ba/F3 cell models. **A,** IC_50_ values of eight selective FGFR inhibitors in viability assays against FGFR3::TACC3 with a WT kinase domain or harboring mutations found in postprogression patient samples. IC_50_ values (nmol/L) are reported as means of ≥3 independent datasets (see Supplementary Table S3). **B,** Immunoblot analysis confirming the ability of erdafitinib to abrogate intracellular signaling in FGFR3::TACC3 WT Ba/F3 cells, while resistance was observed in N540K, V553M, V555L, V555M, and L608V mutants. **C,** Higher concentrations of pemigatinib (100–300 nmol/L) were required to abrogate FGFR3 signaling in FGFR3::TACC3 V553L Ba/F3 compared with erdafitinib (10–30 nmol/L). **D,** Exposing FGFR3::TACC3 WT, N540K, and V555L Ba/F3 cells to erdafinitib corroborated the findings of resistance to erdafinitib, and confirmed the role of “molecular brake disruption” of the N540K mutant. **E** and **F,** Functional characterization of FGFR2 L551F mutation in Ba/F3 cells harboring the *FGFR2::BICC1* transcript. A representative study of a cell viability assay is reported at the top (**E**), and IC_50_ values (nmol/L) are reported at the bottom as means of ≥3 independent datasets (**F**).

In order to evaluate the functional impact of FGFR3 kinase domain mutations on the activity of FGFR inhibitors, we generated Ba/F3 cells with an *FGFR3::TACC3* fusion harboring either N540K, V553L, V553M, V555L, V555M, or L608V mutations. The expression levels of the FGFR3::TACC3 exogenous fusion protein were homogeneous across cell lines (Supplementary Fig. S4). Ba/F3 cells were exposed to increasing doses of the FGFR inhibitors erdafitinib, infigratinib, pemigatinib, rogaratinib, futibatinib, derazantinib, AZD4547, and zoligratinib to determine their corresponding IC_50_ values (Fig. 4A; Supplementary Table S3). LY2874455 had an IC_50_ of 87.5 nmol/L against parental Ba/F3 cells (without *FGFR3::TACC3* fusion), suggesting the drug exerted off-target effects on proliferation. In light of this observation, and considering that LY2874455 is no longer in clinical development, we did not include it in the pharmacologic experiments.

The eight selective FGFR inhibitors had IC_50_ values in the subnanomolar/nanomolar range against *FGFR3::TACC3* WT-driven Ba/F3 cell lines. Each FGFR3 kinase domain mutation conferred a variable increase in the IC_50_ values to FGFR inhibitors. The IC_50_ against the frequent N540K mutant was >100 nmol/L for all the agents with the exception of erdafitinib and futibatinib (5 times lower). Similarly, erdafitinib and futibatinib retained low activity against the gatekeeper V555L/V555M, V553M, and L608V mutations. The V553L mutant appeared to confer a moderate degree of resistance to FGFR inhibitors, as IC_50_ values were close to 10 nmol/L for five inhibitors but at subnanomolar/nanomolar concentrations for erdafitinib, infigratinib, and futibatinib. Of note, this mutation emerged in a patient progressing on pemigatinib (MR406; Fig. 3A and D).

To corroborate our observations, we performed immunoblot analyses with increasing concentrations of erdafitinib in Ba/F3 cells harboring a WT or a mutated *FGFR3::TACC3* construct, in the presence of stimulation with heparin and acidic FGF (aFGF). Analysis of intracellular signaling across models confirmed the viability assays. FGFR3, AKT, and ERK phosphorylation was abrogated after treatment with low concentrations of erdafitinib in the WT model, whereas higher doses were required in the N540K, V553M, V555L, and L608V mutants (Fig. 4B). FGFR3 phosphorylation and intracellular signaling were maintained in the presence of high doses of FGFR inhibitors with the V555M mutation. We confirmed that compared with pemigatinib lower concentrations of erdafitinib were sufficient to abrogate intracellular signaling in the V553L mutant (Fig. 4C). This sustains the hypothesis of an efficient sequential treatment with erdafitinib in case of the emergence of a V553L mutation, which would also be the case with infigratinib and futibatinib according to the viability assays (Fig. 4A).

When Ba/F3 cells having FGFR3::TACC3 WT, FGFR3::TACC3 N540K, and FGFR3::TACC3 V555L constructs were exposed to 30 nmol/L of erdafitinib, the inhibitor was able to abolish the signaling only in the WT model (Fig. 4D). Of note, in the untreated condition, we noticed increased signaling in both mutants, which was especially marked in the N540K mutant in terms of basal ERK phosphorylation, in line with the “molecular brake” nature of the N540K mutation (15, 16).

We also aimed to validate the role of the FGFR2 L551F mutation in conferring resistance to pemigatinib in patient MR904, and to seek alternative agents retaining activity against the mutant. We generated Ba/F3 cells harboring *FGFR2::BICC1* with the L551F mutation, and exposed them to the eight FGFR inhibitors (Fig. 4E and F). In addition to confirming pemigatinib resistance induced by the L551F mutation, our data revealed that erdafitinib and futibatinib could overcome this mutation.

#### Combining FGFR and PIK3CA Inhibition Is Synergic in a Resistant Model of FGFR-Driven Urothelial Cancer Harboring an Activating PIK3CA Mutation

**Figure 5.**
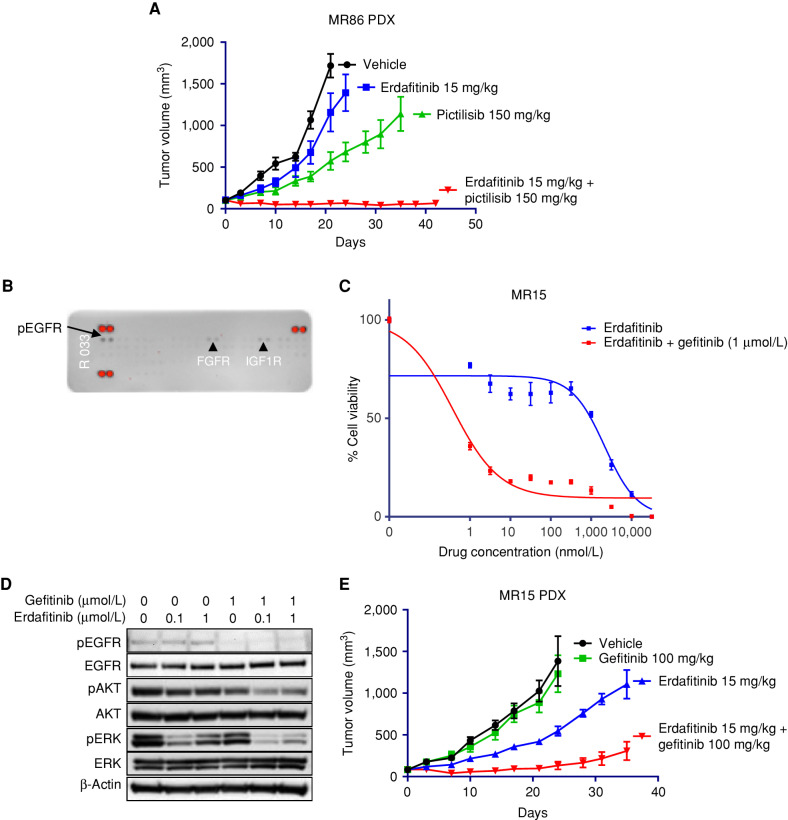
Synergistic effect of combinatorial treatments in patient-derived models of *FGFR*-driven urothelial cancer. **A,** In an MR86 PDX, combined erdafitinib–pictilisib (PI3K inhibitor) administration had synergistic effects leading to prolonged inhibition of tumor growth. **B,** Protein extracts from the MR15 cell line subjected to a phospho-RTK array revealed EGFR activation. **C** and **D,** Concomitant EGFR and FGFR inhibition with gefitinib and erdafitinib was synergic both in viability assays (**C**) and in immunoblot analyses (**D**). **E,** The *in vivo* pharmacologic experiment in the corresponding PDX confirmed the efficiency of the combined treatment strategy.

To elucidate the potential role of PIK3CA E545K in resistance to FGFR inhibitors, we established patient-derived models as previously described (17). In the corresponding MR86 patient-derived xenograft (PDX) model, we evaluated the tumor growth using erdafitinib and pictilisib (PI3K inhibitor) as single agents or in combination. As depicted in Fig. 5A, only the combination treatment was efficient in inhibiting the tumor growth, suggesting that, whereas responses in patients with a PIK3CA preexisting mutation are possible, the PIK3CA E545K mutation contributes to the oncogenic signaling and its targeting seems relevant to maximize tumor response.

#### Nonmutational Resistance Mechanism Identified Using Patient-Derived Models

Patient MR15 suffered from an upper urothelial tumor driven by FGFR3 S249C. At progression on erdafitinib, the tissue biopsy did not reveal any additional genomic alterations compared with the baseline sample. A PDX and patient-derived cell line were established. After confirming that the eight FGFR inhibitors could not restore sensitivity in the patient-derived cell line, we performed a phospho-RTK array and detected hyperphosphorylation of EGFR (Fig. 5B). Although erdafitinib and the EGFR inhibitor gefitinib did not have a significant effect on cell growth inhibition as single agents, the combinatorial treatment showed a synergistic effect in the MR15 cell line (Fig. 5C; Supplementary Fig. S5). Immunoblot analyses confirmed that the double inhibition was required to inhibit intracellular signaling (Fig. 5D). The *in vivo* pharmacologic study confirmed the synergistic effect of combining erdafitinib and gefitinib (not active as single agents) in inhibiting tumor growth in the MR15 PDX (Fig. 5E).

## DISCUSSION

The positive clinical data of FGFR inhibitors in *FGFR*-driven bladder cancer provide the first proof of concept for precision medicine in urothelial cancer. Nevertheless, the ORR of 40% and the median PFS of 5.5 months observed with erdafitinib indicate that there is space for improvement in the field of FGFR-targeted therapies for urothelial malignancies (10). Understanding the mechanisms of resistance to FGFR inhibitors represents a key step in developing new treatment strategies to improve the overall response and the durability of clinical benefit for patients in the setting of molecular-guided therapies.

In this study, we identified both on-target and off-target mechanisms of resistance to FGFR inhibitors in *FGFR*-driven urothelial carcinoma. Our data sustain the complementarity of the information derived from both tissue biopsies and ctDNA. If ctDNA is able to catch the heterogeneity of molecular modifications arising across tumor lesions, tissue biopsy is informative in the case of low ctDNA shedding, especially when disease progression is limited to the thorax, with a mild tumor burden (18, 19). In addition, tissue biopsy allows the establishment of patient-derived models, which are essential tools to further understand resistance and to test new treatments and combination strategies (20). We were not able to identify the resistance mechanism in five out of 21 patients (24%) included in our cohort. Although this proportion of “unknown resistance mechanisms” can be considered low, likely thanks to the availability of multiple sources of molecular analysis at progression on FGFR inhibitors (blood, tissue, and patient-derived models), it remains challenging to systematically characterize the cause of resistance when no explanatory mutational event is detected or when tumor cell–extrinsic factors within the tumor microenvironment contribute to the resistance.

Across our cohort of 19 *FGFR3*-driven urothelial cancers, we identified FGFR3 kinase domain mutations in seven patients at progression on FGFR inhibitors (37%). Of note, the characteristics of the FGFR3 kinase domain at resistance are distinctly different from what has been observed in *FGFR2*-driven cholangiocarcinoma. In patients suffering from cholangiocarcinoma, the occurrence of polyclonal (i.e., multiple) FGFR2 kinase domain mutations is frequently reported at progression on reversible FGFR inhibitors (21–23). In our study, multiple FGFR3 kinase domain mutations were detected only in patient CTC1845 (N540K, V555L, and L608V). The sole availability of ctDNA (lack of tissue biopsy) at the moment of progression on futibatinib in this patient previously treated with erdafitinib precludes the assessment of the potential serial acquisition of the mutations, and their allelic status could not be determined due to ctDNA amplicon size limitation. Pal and colleagues have previously reported the detection of V555L/M and L608V in four patients at progression on erdafitinib, but only one case had a polyclonal presentation (V555M and L608V; ref. 11). In addition to these two mutations, we reported for the first time additional mutations occurring in the FGFR3 kinase domain mutation at progression on selective inhibitors: N540K, V553L, V553M, and E587Q. With the exception of the latter, all the other mutational events have been reported in their corresponding positions of FGFR2 (N550K, V563L, V565I/L/F, L618V, according to the NM_001144913.1 transcript), suggesting that these kinase domain conserved residues are resistance hotspots in the FGFR3 kinase domain as well (21–23). Whereas it is difficult to derive the incidence of precise mutations from our study with a limited sample size, the gatekeeper residue FGFR3 V555 and the molecular brake N540 could represent relevant targets to consider for the development of novel inhibitors due to their higher frequency and resistance pattern.

Although confirming the role of FGFR3 kinase domain mutations in conferring resistance to the inhibitors, our functional studies were not informative in recognizing agents able to overcome on-target resistance. The only exception was represented by the potential role of erdafitinib, infigratinib, and futibatinib against the V553L mutation acquired in the post-pemigatinib sample. Of note, among reversible FGFR inhibitors, erdafitinib yielded the lowest IC_50_ values against both WT and mutated *FGFR3:TACC3* Ba/F3 cell lines, potentially explaining its better outcomes observed in clinical trials.

The immunoblots and cell viability analyses highlighted the differential contribution of the molecular brake (N540K) and gatekeeper (V555L) mutation in conferring resistance. V555L, thought to interfere with drug binding (24), boosts intracellular signaling as well, mainly represented by ERK phosphorylation. This latter was even more marked in the presence of N540K, in line with the “molecular brake” function of the N540 residue (15, 16). From this point of view, novel inhibitors will need to harbor higher potency, and their design should allow them to circumvent the steric clash induced by gatekeeper (and other) mutations.

The functional evidence generated regarding the FGFR2 L551F mutation also suggested it could be therapeutically targeted in *FGFR2*-driven tumors. In our patient MR904, the mutation emerged at progression on pemigatinib, and Varghese and colleagues reported a similar finding at progression on infigratinib in a patient affected by an *FGFR2*-driven cholangiocarcinoma (one additional patient developed L551F in the context of polyclonal FGFR kinase domain mutations; ref. 21). The FGFR2 L551F mutation was not previously characterized, and our data suggest that L551F, while engendering resistance to pemigatinib and infigratinib, could be overcome by futibatinib and erdafitinib.

In addition to on-target alterations responsible for resistance, we identified recurrent events affecting the PI3K–mTOR pathway at resistance to FGFR inhibitors in urothelial tumors, concomitantly or not with FGFR3 kinase domain mutations. In our population and in molecular landscape studies of urothelial tumors, *PIK3CA* and *TSC1/2* alterations are not mutually exclusive with *FGFR3* in baseline samples (1–5, 25). Nevertheless, their enrichment in postprogression samples strongly implicates their involvement in resistance. As supported by models derived from patient MR86, a baseline activating mutation in *PIK3CA* may not be fully capable of conferring resistance but may represent a suitable target to improve responses. The observed alterations in the PI3K–mTOR pathway in clinical samples from this study have been shown to be responsible for progression on targeted therapies in other oncogene-driven diseases and suitable targets for specific inhibition (26–31).

The establishment of the patient-derived cell line and PDX of patient MR15 illustrates the relevance of such models in providing critical elements in addressing off-target, nongenomic mechanisms of resistance and enables the development of new strategies for treatment paradigms. Of note, EGFR bypass activation has been reported as an early event in the setting of FGFR inhibition in *FGFR3*-altered urothelial cancer and in *FGFR2*-driven cholangiocarcinoma, suggesting the potential of EGFR targeting in progressive *FGFR*-driven diseases (32, 33).

This study has several limitations. Although being the largest study on the subject thus far, the limited sample population precludes the generalization of our findings. Despite the availability for the majority of the patients, pretreatment sequencing analyses could not be performed systematically. The lack of a matched collection of postprogression tissue samples and ctDNA in some patients limits the depth of the molecular insights we could obtain. Despite our systematic attempt in establishing patient-derived models, the relatively low success rate limited the identification of nongenetic-driven resistance mechanisms. Finally, we did not validate functionally the role of *TSC1/2* mutations or their allelic status.

Altogether, our study represents the first systematic approach toward understanding resistance to FGFR inhibitors in *FGFR*-driven urothelial cancer. The evidence generated on both on-target and off-target molecular events responsible for resistance will serve as the backbone to address the development of new therapeutic strategies to improve patient outcomes.

## METHODS

### Patients and Treatments

To be included in this study, patients had to satisfy the following criteria: (i) diagnosis of an advanced urothelial cancer; (ii) treatment with a selective FGFR inhibitor due to the detection of a molecular alteration in one of the *FGFR* family members; and (iii) availability of postprogression tissue and/or ctDNA available for molecular analyses.

Patients started FGFR inhibitor treatment between 2013 and 2021. Patients were treated in the setting of clinical trials or compassionate use programs allowing treatment with FGFR inhibitors on the basis of molecular selection. Disease response was measured according to RECIST, and PFS was calculated from the date of the targeted inhibitor start to the day of radiologic evidence of progression.

The molecular analyses were performed within institutional studies ongoing at Gustave Roussy whose aim is the molecular characterization of tumors: MATCH-R (NCT02517892; ref. 17), MOSCATO (NCT01566019; ref. 34), STING-UNLOCK (NCT04932525), and CTC (NCT02666612).

All patients participating in the mentioned studies were fully informed and signed written informed consent. The studies have been approved by the ethics committee at Institut Gustave Roussy and the French National Agency for Medicines and Health Products Safety (ANSM), and are being conducted in accordance with the Declaration of Helsinki.

The objectives of the MOSCATO and MATCH-R studies are to address patients to targeted treatments according to molecular alterations and to assess molecular mechanisms of resistance, respectively. These studies initially focused on molecular analysis performed on tissue biopsy, as ctDNA technologies were not commonly used and blood/plasma samples were not collected (see MOSCATO patient M521, and MATCH-R patients MR15 and MR86). On the other hand, CTC and STING-UNLOCK were developed to study molecular alterations from blood samples (exclusively for CTC, including tissue biopsy for STING-UNLOCK). The availability of postprogression samples relied on the precise study in which individual patients were enrolled.

### Molecular Analyses

Postprogression tumor, when possible, underwent WES with or without concomitant RNA-seq. The main limitation of WES/RNA-seq performance was the proportion of tumor cells ≥30% in the tissue sample. In cases in which the proportion of tumor cells was between 10% and 30%, molecular analyses with targeted NGS panels (Mosc-3, Oncomine v3) were performed. For WES, the mean coverage was 140×.

ctDNA analyses were performed with Illumina or Foundation Medicine liquid biopsy panels. Importantly, all ctDNA sequencing had complete coverage of the kinase domains of *FGFR* genes. Briefly, extracted circulating free DNA from patient samples was processed through a library preparation that includes the addition of unique molecular identifiers, followed by a hybrid capture–based workflow and high-depth sequencing. For each patient with longitudinal ctDNA assessment, only analyses performed with the same platform were reported.

### Site-Directed Mutagenesis

Lentiviral vectors expressing *FGFR3*::*TACC3* fusions were created using the pLenti6/V5 directional TOPO Cloning Kit (#K495510, Thermo Fisher Scientific) according to the manufacturer's instructions. Point mutations in the FGFR3 kinase domain of the *FGFR3*::*TACC3* fusion were introduced using the QuickChange XL Site-Directed Mutagenesis Kit (#200516, Agilent) according to the manufacturer's protocol and using the following primers:

FGFR3 N540K forward (F) CAAAAACATCATCAAGCTGCTGGGCGCCTGC
FGFR3 N540K reverse (R) GCAGGCGCCCAGCAGCTTGATGATGTTTTTG
FGFR3 V553L F CGCAGGGCGGGCCCCTGTACTTGCTGGTGGAGTACGCGGCC
FGFR3 V553L R GGCCGCGTACTCCACCAGCAAGTACAGGGGCCCGCCCTGCG
FGFR3 V553M F CGCAGGGCGGGCCCCTGTACATGCTGGTGGAGTACGCGGCC
FGFR3 V553M R GGCCGCGTACTCCACCAGCATGTACAGGGGCCCGCCCTGCG
FGFR3 V555L F GCCCCTGTACGTGCTGCTGGAGTACGCGGCCAA
FGFR3 V555L R TTGGCCGCGTACTCCAGCAGCACGTACAGGGGC
FGFR3 V555M F GCCCCTGTACGTGCTGATGGAGTACGCGGCCAA
FGFR3 V555M R TTGGCCGCGTACTCCATCAGCACGTACAGGGGC
FGFR3 L608V F AGGTGGCCCGGGGCATGGAGTACGTGGCCTCCCAGAAGTGCATCCAC
FGFR3 L608V R GTGGATGCACTTCTGGGAGGCCACGTACTCCATGCCCCGGGCCACCT

The same procedure was used to create lentiviral vectors expressing *FGFR2::BICC1*, and the primers used for creating L551F mutations were as follows:

FGFR2 L551F F GGGAAACACAAGAATATCATAAATTTTCTTGGAGCCTGCACACAGGATG
FGFR2 L551F R CATCCTGTGTGCAGGCTCCAAGAAAATTTATGATATTCTTGTGTTTCCC

### Allelic Distribution of FGFR3 Mutations

To assess the allelic status of FGFR3 S249C and V553L in patient MR406, mRNA obtained from the tissue biopsy underwent retro-transcription, and a PCR spanning the two mutated residues was performed on cDNA. Amplicons were subcloned into the pCR2.1-TOPO vector (Invitrogen) according to the manufacturer's protocol. Individual cDNA was sequenced by Sanger sequencing to determine the *cis*/*trans* status of mutations.

### Development of PDXs in Mice and *In Vivo* Pharmacologic Studies

All animal procedures and studies have been approved by the French Ministry of “Education nationale, de l'Enseignement supérieur et de la Recherche” (APAFIS#2790-2015112015055793 and APAFIS#2328-2015101914074846). Fresh tumor fragments were implanted in the subrenal capsule of 6-week-old female NOD/SCID gamma (NSG) mice obtained from Charles River Laboratories. PDX-bearing NSG mice were treated with gefitinib [100 mg/kg once daily (qd) in 0.5% methylcellose, 0.5% Tween 80] or erdafitinib [15 mg/kg qd in 10% HP-beta-CD (hydroxypropyl beta cyclodextrin)] or pictilisib (150 mg/kg qd in 0.5% methylcellose, 0.5% Tween 80) alone or in combination by oral gavage. Eight mice per group were treated for up to 40 days, and tumor volume and mouse weight were measured twice per week.

### Cell Lines

Parental Ba/F3 cells were purchased from DSMZ (ref. ACC 300) and cultured in DMEM 10% FBS in the presence of IL3 (0.5 ng/mL). Ba/F3 cells were infected with lentiviral constructs, as reported previously (35), to express the *FGFR3::TACC3* or the *FGFR2::BICC1* fusions with a WT kinase domain or with the mentioned kinase domain mutations. Infected Ba/F3 cells harboring FGFR fusions were selected in the presence of blasticidin (14 mg/mL) and IL3 (0.5 ng/mL) until recovery, and a second selection by culturing the cells in the absence of IL3 was then performed. *FGFR2*/*3* fusions and corresponding kinase domain mutations were confirmed on the established cell lines by Sanger sequencing.

After several attempts, we were not able to establish IL3-independent Ba/F3 cells driven by FGFR3 S249C or FGFR3 Y373C mutations. Previous experience showed that FGFR2 cannot drive IL3-independent proliferation in the same way as FGFR1 (16), and we envisage that FGFR3 mutations occurring in the extracellular domain, oncogenic drivers in human tumors, are not sufficient to fulfill this function in Ba/F3 models in isolation.

Patient-derived cell lines (MR15) were developed from PDX samples by enzymatic digestion with a tumor dissociation kit (ref. 130-095-929, Miltenyi Biotec) and mechanical degradation with the gentleMACS dissociator. Cells were cultured with DMEM/F-12 GlutaMAX 10% FBS and 10% enriched with hydrocortisone 0.4 mg/mL, cholera toxin 8.4 ng/mL, adenine 24 mg/mL, and ROCK inhibitor 5 mmol/L (Y-27632, S1049 Selleckchem) until a stable proliferation of tumor cells was observed, as described previously (36). Culture media were then transitioned to DMEM and cultured in the presence of erdafitinib 300 nmol/L to 1 mmol/L.

As for cell verification, we confirmed that engineered Ba/F3 as well as MR15 patient-derived cell line harbored the corresponding *FGFR* fusion and mutations by RT-PCR and Sanger sequencing. The cells were not tested for *Mycoplasma* contamination, but cells were not maintained in culture for more than 2 months after establishment or thawing.

### Reagents

Erdafitinib, infigratinib, pemigatinib, futibatinib, derazantinib, AZD4547, zoligratinib, and LY2874455 were purchased from Selleck Chemicals. Rogaratinib was purchased from MedChemExpress.

### Cell Viability Assays and Immunoblots

Cell viability assays were performed in 96-well plates using the CellTiter-Glo Luminescent Cell Viability Assay (G7570, Promega). For Ba/F3, we seeded 4,000 cells/well and we treated cells for 48 hours. For the MR15 patient-derived cell line, we seeded 3,000 cells/well and treated cells for 5 days.

Ba/F3 cells with *FGFR3::TACC3* fusion with a WT or a mutated kinase domain were treated for 24 hours with the corresponding doses of FGFR inhibitors in the presence of heparin 25 μg/mL (Sigma-Aldrich, ref. H3149) and aFGF 50 ng/mL (R&D Systems, ref. 232-FA-025/CF), as previously reported (37).

For Western blot assays, the following antibodies were purchased from Cell Signaling Technology: pFGFR (3471S), pAKT (4060S), AKT (4691S), pERK (9101S), ERK (9102S), pS6 (4858S), S6 (2217S), and BIM (2933S). Anti-FGFR3 (ab133644) and anti-pEGFR (ab5644) antibodies were purchased from Abcam, anti-EGFR (sc-03) antibody was purchased from Santa Cruz Biotechnology, and anti–β-actin (A1978) antibody was purchased from Sigma-Aldrich.

### Data Availability

WES/RNA-seq raw data files from this study are deposited at the European Genome–phenome Archive (EGA) using the accession code EGAS00001007335. Access to this shared dataset is controlled by the institutional Data Access Committee, and requests for access can be sent to the corresponding author. Further information about EGA can be found at https://ega-archive.org/. Any additional information required to reanalyze the data reported in this article is available upon request from the corresponding author.

## Supplementary Material

Supplementary Table 1 Patient population, treatment outcomes and post-progression sample availability.

Supplementary Table 2 Systemic treatment received by patients prior to selective FGFR inhibitors, according to the detection of FGFR kinase domain mutations and/or PI3K/mTOR pathway alterations.

Supplementary Table 3 IC50 values of Ba/F3 cells harboring FGFR3:TACC3 mutants exposed to eight selective FGFR inhibitors.

Supplementary Figure 1 RNAseq analysis from tissue biopsies of 39 advanced urothelial cancers.

Supplementary Figure 2 Documentation of the allelic status in cis of FGFR3 S249C and V553L mutations from MR406 tissue biopsy.

Supplementary Figure 3 Acquisition of mutations in PI3K/TSC1 pathway at progression to FGFR inhibitors.

Supplementary Figure 4 Expression of FGFR3:TACC3 was homogeneous across Ba/F3 cell lines.

Supplementary Figure 5 Synergic effect of gefitinib and erdafitinib in MR15 cell line.
