# Resistance to selective FGFR inhibitors in *FGFR-driven* urothelial cancer

**DOI:** 10.1158/2159-8290.CD-22-1441

**Published:** 2023-06-28

**Authors:** Francesco Facchinetti, Antoine Hollebecque, Floriane Braye, Damien Vasseur, Yoann Pradat, Rastislav Bahleda, Cédric Pobel, Ludovic Bigot, Olivier Déas, Juan David Florez Arango, Giorgia Guaitoli, Hayato Mizuta, David Combarel, Lambros Tselikas, Stefan Michiels, Sergey I Nikolaev, Jean-Yves Scoazec, Santiago Ponce-Aix, Benjamin Besse, Ken A Olaussen, Yohann Loriot, Luc Friboulet

**Affiliations:** 1 Université Paris-Saclay, Gustave Roussy, Inserm U981, Villejuif, France; 2 Département d'Innovation Thérapeutique (DITEP), Gustave Roussy, Villejuif, France; 3 Département de Médecine Oncologique, Gustave Roussy, Villejuif, France; 4 Medical Biology and Pathology Department, Gustave Roussy, Villejuif, France; 5 AMMICa UAR3655/US23, Gustave Roussy, Villejuif, France; 6 Université Paris-Saclay, CentraleSupélec, MICS lab, Gif-Sur-Yvette, France; 7 XenTech, Evry, France; 8 PhD Program Clinical and Experimental Medicine, University of Modena and Reggio Emilia, Modena, Italy; 9 BIOTHERIS, Department of Interventional Radiology, Gustave Roussy, Université Paris-Saclay, Villejuif, France; 10 Université Paris-Saclay, Inserm, CESP, Villejuif, France; 11 Gustave Roussy, Office of Biostatistics and Epidemiology, Villejuif, France; 12 Université Paris-Saclay, Faculté de Médecine, Le Kremlin Bicêtre, France

**Keywords:** Bladder cancer, upper urothelial tumor, FGFR, resistance, targeted treatment

## Abstract

Several FGFR inhibitors are approved or in clinical development for the treatment of *FGFR-driven* urothelial cancer, and molecular mechanisms of resistance leading to patient relapses have not been fully explored. We identified 21 *FGFR*-driven urothelial cancer patients treated with selective FGFR inhibitors and analyzed post-progression tissue and/or circulating tumor DNA (ctDNA). We detected single mutations in the *FGFR* tyrosine kinase domain in seven (33%) patients (FGFR3 N540K, V553L/M, V555L/M, E587Q; FGFR2 L551F) and multiple mutations in one (5%) case (FGFR3 N540K, V555L, L608V). Using Ba/F3 cells, we defined their spectrum of resistance/sensitivity to multiple selective FGFR inhibitors. Eleven (52%) patients harbored alterations in the PI3K-mTOR pathway (n = 5 *TSC1/2*, n = 5 *PIK3CA*, n = 1 *NF2*, n = 1 *PTEN).* In patient-derived models, erdafitinib was synergic with pictilisib in the presence of PIK3CA E545K, while erdafitinib-gefitinib combination was able to overcome bypass resistance mediated by EGFR activation.

## Introduction

Molecular alterations in fibroblast growth factor receptor (*FGFR*) gene family are frequent events in urothelial cancer. *FGFR3* mutations, a hallmark of low-grade, non-muscle invasive bladder cancer, are present in 12-22% of high-grade, muscle invasive tumors (1–4). Upper tract urothelial carcinomas, representing less than 10% of urothelial cancers, harbor *FGFR3* mutations in up to 60% of high-grade cases (1,4,5). FGFR3 activating mutations mainly occur in the extracellular domain of FGFR3 (S249C, R248C and Y373C hotspots) (3–5). Oncogenic *FGFR3* fusions (with *TACC3* as the most frequent partner gene) are found in up to 2-3% of bladder cancer, and detected in upper tract urothelial carcinoma as well (2,4–6). Activating alterations in other *FGFR* genes, although rare, are also reported in urothelial tumors (7,8).

Oncogenic activating *FGFR* alterations in urothelial carcinomas represent an opportunity for targeted therapeutic intervention with selective FGFR inhibitors. In a phase II trial leading to FDA approval, 101 patients with *FGFR3* mutations or *FGFR2/3* rearrangements treated with erdafitinib at the starting dose of 8 mg daily, the objective response rate (ORR) and median progression-free survival (PFS) were 40% and 5.5 months, respectively (9,10). Two additional reversible inhibitors, infigratinib and pemigatinib, and the irreversible inhibitor futibatinib led to slightly inferior outcomes in FGFR-selected populations of patients with urothelial cancer, acknowledging that futibatinib data were mainly obtained from a population pre-treated with FGFR inhibitors (11–13). Recently, in a phase II trial the reversible inhibitor rogaratinib did not show superiority to chemotherapy in pretreated patients selected for FGFR1/3 mRNA overexpression. Among the 21 patients with *FGFR3* mutations or fusions randomized to receive rogaratinib, responses were observed in 11 cases (ORR 52.4%); 4 out of 15 patients with FGFR3 molecular alterations treated with chemotherapy achieved disease response (ORR 26.7%) (14).

In line with other oncogene-driven diseases, primary and acquired resistance limit the efficacy of FGFR inhibitors in urothelial carcinomas. Evidence regarding mechanisms of resistance to FGFR inhibitors is limited to the analysis of 22 ctDNA samples at progression to infigratinib. In four patients, acquired mutations in the FGFR3 tyrosine kinase domain were detected (FGFR3 V555L, V555M and L608V, according to the NM-000142.4 transcript) (11).

Here we present a molecular and functional study of resistance to selective FGFR inhibitors in patients with *FGFR*-driven urothelial cancer. FGFR tyrosine kinase domain mutations and off-target molecular activations were frequently observed. According to the resistance mechanisms recorded, we evaluated adaptive *(i.e.* alternative FGFR inhibitors) and combinatorial treatment strategies in preclinical models, providing potential future guidance for clinical application.

## Results

### Molecular landscape of advanced/metastatic urothelial cancers

We collected clinical and molecular data for a total of 56 advanced/metastatic urothelial cancer patients (n = 39 bladder, n = 15 upper urothelial tract, n = 2 with both localizations). 38 patients had both whole exome sequencing (WES) and RNA sequencing (RNAseq) performed on tissue biopsy, 9 WES only, 1 with RNAseq only; 8 patients had a targeted next-generation sequencing (NGS) panel on circulating tumor DNA (ctDNA) and/or tissue biopsy.

**Figure 1:**
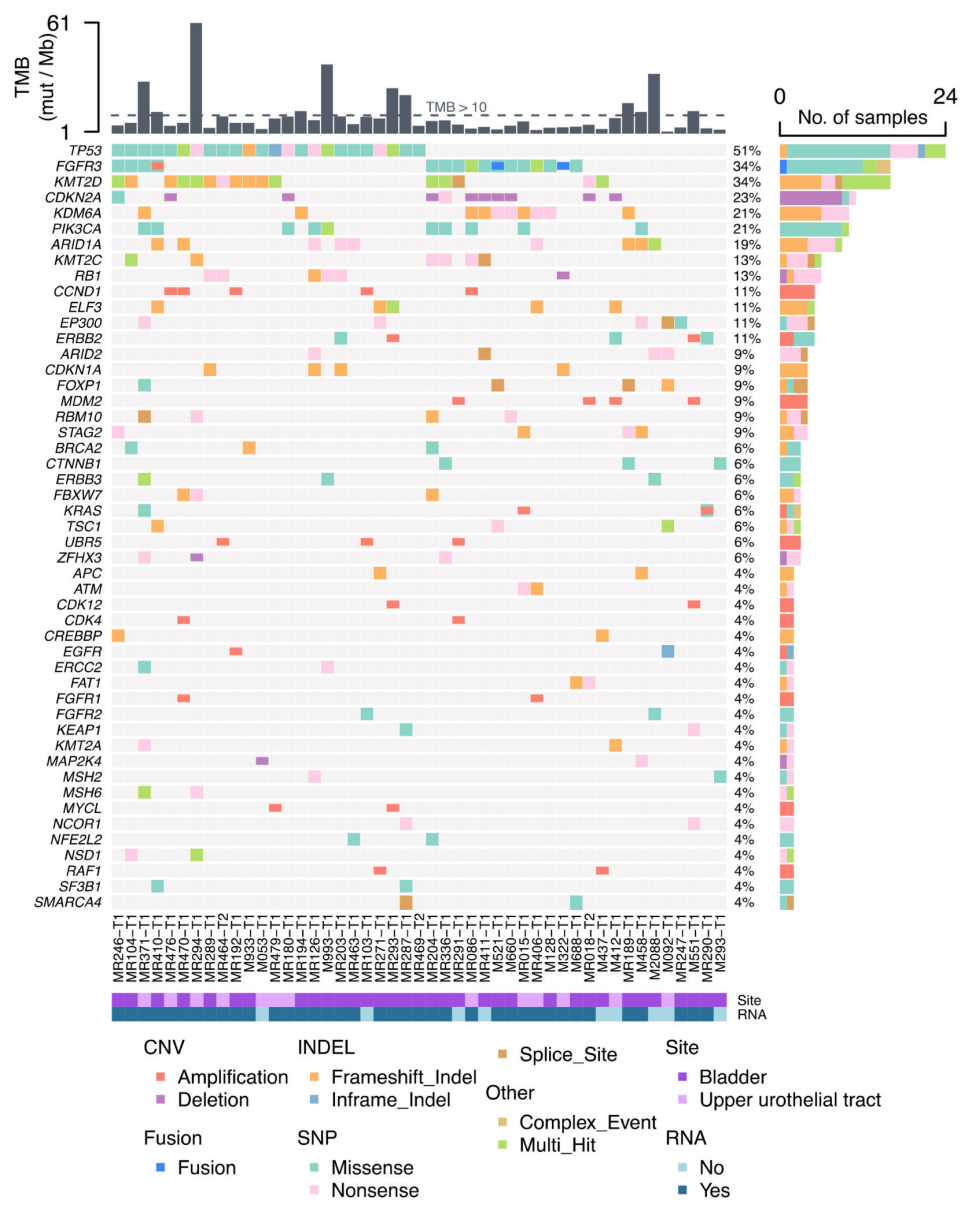
Mutational landscape of urothelial cancers included in MATCH-R or MOSCATO studies, with WES +/- RNAseq performed. TMB: Tumor mutation burden; mut: Mutations; Mb: Megabase; No.: Number; CNV: Copy number variation; SNP: Single nucleotide polymorphism; INDEL: Insertion/deletion.

The assessment of the mutational features has been the main objective of seminal studies shedding light on the frequency of *FGFR3* alterations and other molecular features in urothelial carcinomas, focusing on localized diseases (2,4,5). We therefore performed a global mutational landscape analysis using tumor samples from the 47 urothelial cancer patients with advanced/metastatic disease and WES available (Figure 1). The most frequently altered genes were *TP53* (51%), *FGFR3* (34%), and *KMT2D* (34%). Taking into account the limitation of evaluating expression profiles from bulk RNAseq analyses of tissue biopsies, with variable tumor/stroma cellularity, we investigated differential expression of genes involved in the main signaling pathways in the 39 samples with RNAseq available. No clear pattern was highlighted between *FGFR3*-driven and non *FGFR3*-driven patient samples with the exception of FGFR3 mRNA (Supplementary Figure 1A). Analyzing specifically FGFR family member expression levels, we did indeed reveal a strong significant overexpression of FGFR3 mRNA in *FGFR3*-driven samples compared to *FGFR3* wildtype ones (Supplementary Figure 1B). By contrast, only a slight decrease was observed for FGFR1 and FGFR4 mRNA and no difference for FGFR2 expression.

### Patients progressing on selective FGFR inhibitors

**Figure 2:**
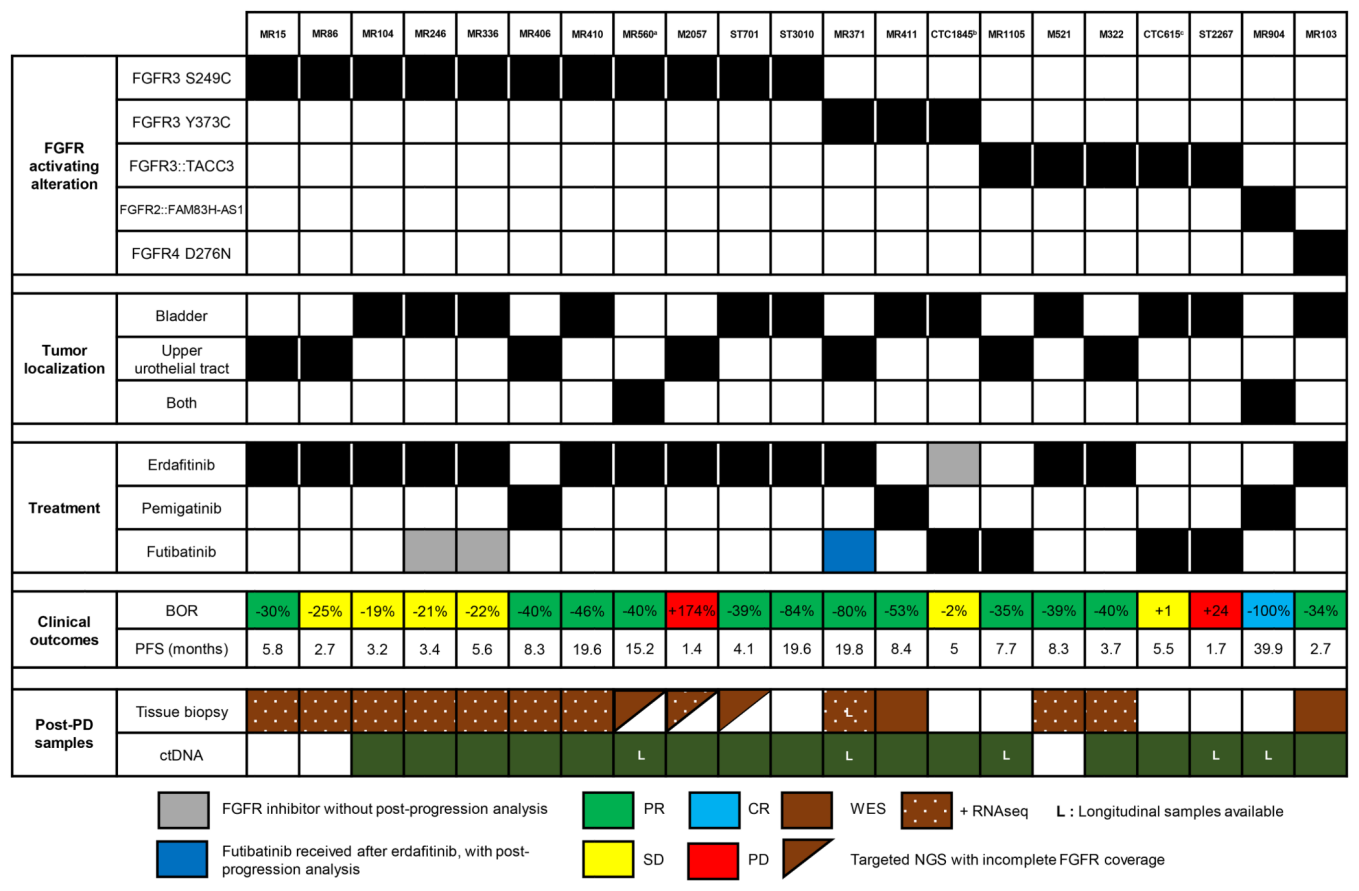
Overview of urothelial cancer patients progressing on FGFR inhibitors, treatment outcomes and post-progression sample availability. Granular view at the individual patient-level. ^a^ Patient MR560 received erdafitinib combined with a PD-1 inhibitor. ^b^ Patient CTC1845 was treated with a sequence of erdafitinib-futibatinib (with intervening immunotherapy between the two FGFR inhibitors) and ctDNA sample was collected at progression on futibatinib. ^c^ Patient CTC615 received pazopanib before futibatinib. CR: Complete response; PR: Partial response; SD: Stable disease; PD: Progressing disease; ctDNA: Circulating tumor DNA; BOR: Best objective response; PFS: Progression-free survival; WES: Whole exome sequencing; RNAseq: RNA sequencing; NGS: Next-generation sequencing.

We then focused on the 21 patients treated with selective FGFR inhibitors; 12 and 7 had bladder and upper tract tumors respectively, and in 2 additional cases both localizations were present. Eleven cases harbored an FGFR3 S249C mutation, 5 an *FGFR3:TACC3* fusion and 3 displayed an FGFR3 Y373C mutation. One patient (MR904) harbored an *FGFR2::FAM83H-AS1* rearrangement and one (MR103) an FGFR4 D276N mutation (Figure 2 and Supplementary Table 1).

Post-progression samples were collected on erdafitinib (n = 14), futibatinib (n = 4) or pemigatinib (n = 3). As best objective responses, we observed one complete response (CR), 12 partial responses (PR), 6 stable diseases (SD) and 2 progressive diseases (PD). Of note, all patients were previously treated for advanced disease, and the majority received the FGFR inhibitor as second treatment line (range 2^nd^-6^th^), but no clear correlation between the type of cytotoxic agents received by patients prior to FGFR inhibitors and molecular alterations could be evidenced (Supplementary Table 2).

12 patients had both post-progression tissue biopsy and ctDNA, 6 had ctDNA only and 3 tissue only. Four patients received futibatinib after having progressed to either erdafitinib or pemigatinib. Longitudinal analysis was possible for 5 patients thanks to additional ctDNA samples, including one patient with tissue molecular profiles obtained both at erdafitinib and futibatinib progression. Overall, we analyzed 39 post-progression samples: 16 post-progression tissue analysis and 23 ctDNA samples.

### Molecular findings at progression on selective FGFR inhibitors in urothelial cancer

#### FGFR kinase domain mutations

**Figure 3:**
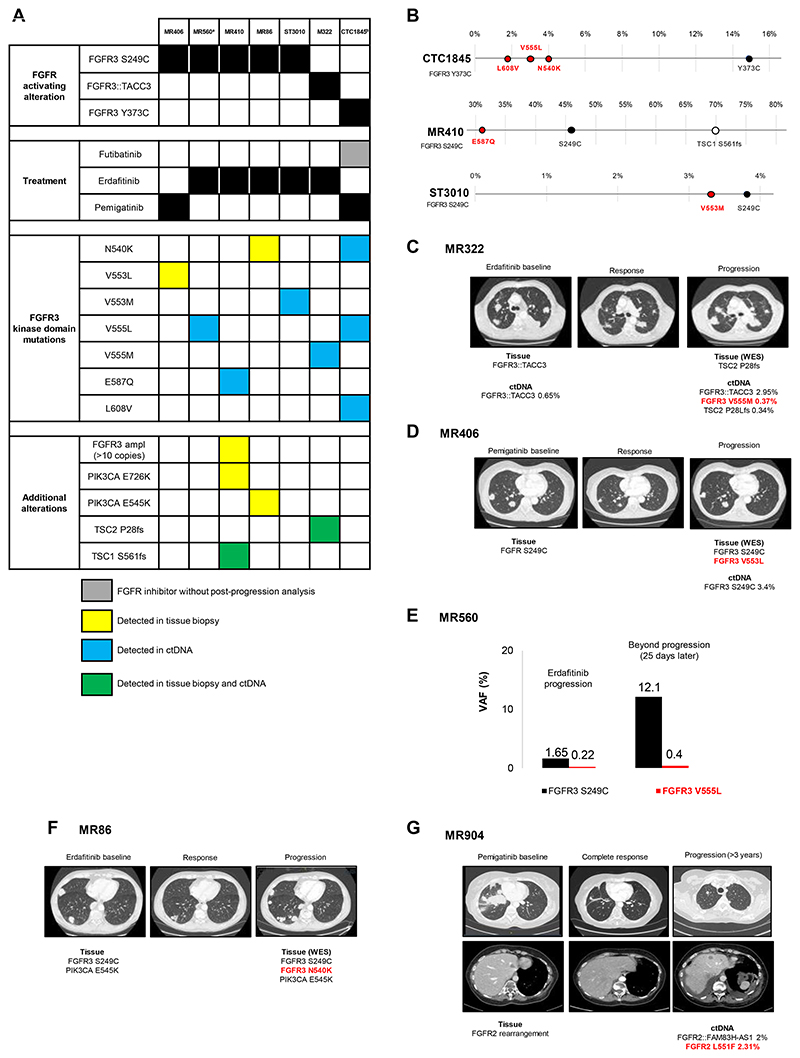
FGFR kinase domain mutations detected at progression to FGFR inhibitors in urothelial cancer. **A:** Heatmap representing individual patient data among the cohort of *FGFR3-driven* urothelial cancers. **B-G:** Molecular and radiological findings. Mutations and fusions in ctDNA are reported as variant allele frequency (VAF). ^a^ Patient MR560 received erdafitinib combined with a PD-1 inhibitor. ^b^ Patient CTC1845 was treated with a sequence of erdafitinib-futibatinib (with intervening immunotherapy between the two FGFR inhibitor) before ctDNA sample was collected at progression on futibatinib. NA: Not assessable; ctDNA: Circulating tumor DNA; ampl: Amplification; WES: whole exome sequencing; NGS: Next-generation sequencing.

In the cohort of 19 *FGFR3*-driven urothelial cancers who progressed on erdafitinib, pemigatinib or futibatinib, FGFR3 kinase domain mutations were detected in 7 patients (37%, Figure 3A). Sequencing data of pre-treatment tissue biopsy and/or ctDNA was available for 5 out of 7 patients (71%), and no pre-existing FGFR3 kinase domain mutation was detected in those baseline samples. Of note, the activating *FGFR3* alterations were detected in all ctDNA and tissue samples available at progression. In 6 patients, we detected single mutations in the FGFR3 kinase domain in either ctDNA or tissue biopsy: N540K, V553L, V553M, V555L, V555M, and E587Q (Figure 3A). Three concomitant FGFR3 kinase domain mutations (N540K, V555L, L608V), in addition to the activating Y373C, were identified in ctDNA of patient CTC1845 (Figure 3A-B) who progressed on futibatinib after having received erdafitinib (PR -67%, PFS 8.6 months). The allelic status (cis/trans configuration) of these four FGFR3 mutations could not be assessed due to ctDNA amplicon size limitation.

Patient MR410 had a FGFR3 E587Q mutation detected in ctDNA only at progression to erdafitinib, a TSC1 S561fs alteration detected both in tissue and in liquid biopsies, while *FGFR3* amplification and PIK3CA E726K mutation were found in the tissue biopsy only (Figure 3B).

In the erdafitinib “baseline” ctDNA sample of patient M322 we observed an *FGFR3::TACC3* fusion, later detected at higher variant allele frequency (VAF) in the post-progression liquid biopsy, together with an FGFR3 V555M mutation and a *TSC2* frameshift event (P28Lfs) (Figure 3C). In line with limited ctDNA shedding in cases of intrathoracic-only disease, the FGFR3 V553L mutation was only detected in the tissue biopsy and not in the ctDNA of patient MR406, who experienced mild, thoracic-only progression to pemigatinib (Figure 3D). Given the availability of mRNA from the tissue biopsy, we performed TOPO-TA cloning and observed FGFR3 S249C and V553L mutations on the same allele, confirming that the mutational events arose *in cis* with the driver mutation (Supplementary Figure 2).

The VAF increase of both FGFR3 S249C and V555L reflected the progression during erdafitinib treatment in patient MR560 (Figure 3E).

For patient MR86, an FGFR3 N540K mutation was detected in tissue biopsy, in addition to the driver mutation FGFR3 S249C and a PIK3CA E545K already existing in the pre-erdafitinib sample (Figure 3F).

Additionally, upon detection of an *FGFR2* rearrangement in one urothelial cancer patient (MR904), third-line pemigatinib resulted in a complete response (Figure 3G). Upon progression (after 3 years and 4 months), ctDNA analysis revealed the fusion partner *(FGFR2::FAM83H-AS1)* as well as an FGFR2 L551F mutation (according to NM -022970.3 transcript).

#### Molecular alterations in the PI3K-mTOR pathway

In 11 out of 19 patients (58%) with *FGFR3*-driven urothelial carcinoma, we detected an alteration in the PI3K-mTOR pathway in post-progression and/or in baseline samples (Table 1).

In five samples a *PIK3CA* activating mutation was detected at progression, but in three cases the *PIK3CA* alteration was present before the treatment with FGFR inhibitors (MR15, MR86 and ST2267). MR15 and MR86 achieved clinical responses on erdafitinib before progression. MR86 acquired an FGFR3 N540K mutation, while an EGFR hyperphosphorylation (determined by a phospho-receptor tyrosine kinase array) was potentially explaining the resistance for MR15 (see Section 2.4.3). By contrast patient ST2267, with a baseline PIK3CA H1047R tumor mutation, experienced primary resistance to futibatinib. For the patient MR336, the PIK3CA E545K mutation was acquired during treatment with erdafitinib, which potentially conferred the primary resistance to futibatinib (Supplementary Figure 3A).

In addition to *PIK3CA* mutations, *TSC1/2* inactivating mutations were also frequently detected (5/19) in patients progressing on FGFR inhibitors (Table 1). MR410 had a TSC1 S561fs mutation detected in both post-progression (erdafitinib) tissue and ctDNA (VAF 69.96%) concurrent with PIK3CA E726K and FGFR3 E589Q (VAF 31.12%) mutations.

A TSC2 P28Lfs emerged in patient M322, a TSC1 Q865* in patient M521, a TSC1 Q830* in patient ST701 at progression to erdafitinib, and a TSC1 A186fs in patient MR1105 at futibatinib progression (Supplementary Figure 3B).

Patient MR246, with an FGFR3 S249C-driven bladder cancer who ultimately progressed on erdafitinib, an NF2 frameshift event was detected by ctDNA together with an FGFR2 V517M mutation.

Finally, a PTEN C136fs mutation was detected in a patient with primary resistance to erdafitinib (M2057).

The frequency of these mutations arising in tumor suppressor genes suggests they should be considered as potential targets for overcoming treatment resistance.

### Functional analyses of molecular alterations and overcoming therapeutic strategies

#### FGFR kinase domain mutations confer resistance to selective FGFR inhibitors

**Figure 4:**
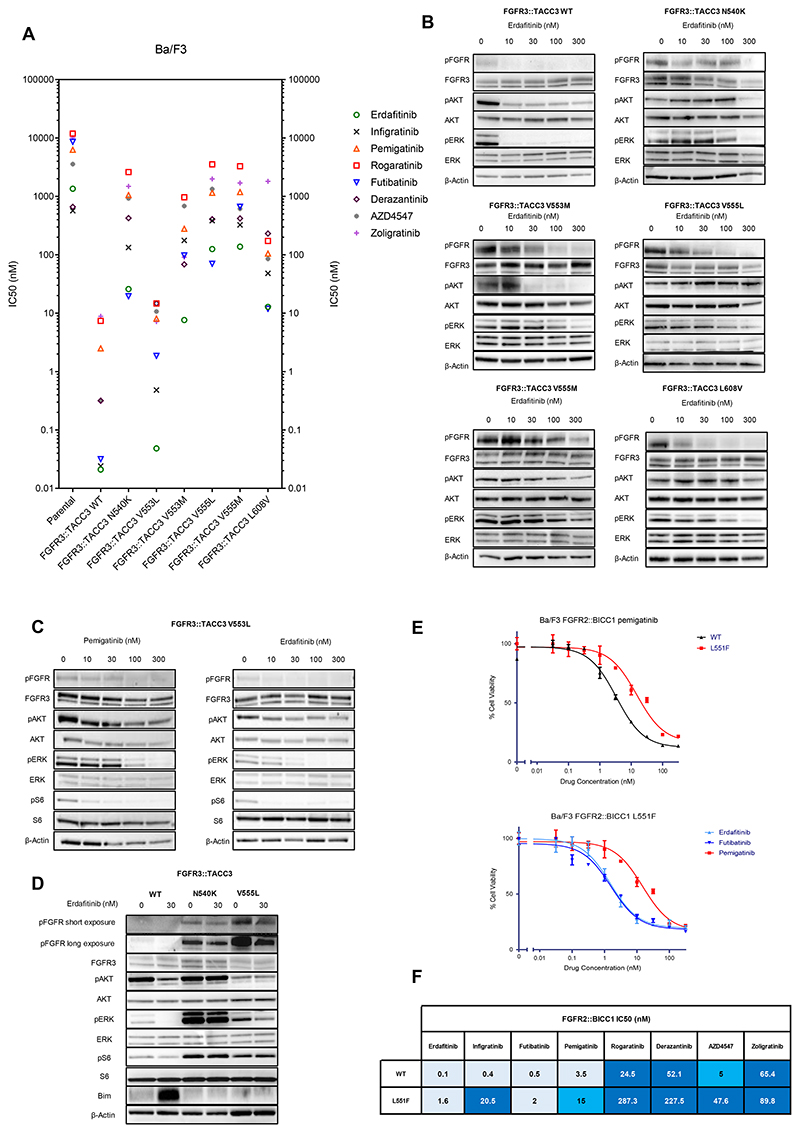
Functional characterization of FGFR2/3 kinase domain mutations in Ba/F3 cell models. **A:** IC50 values of 8 selective FGFR inhibitors in viability assays against *FGFR3::TACC3* with a WT kinase domain, or harboring mutations found in post-progression patients’ samples. IC50 values (nM) are reported as means of ≥ 3 independent data sets (see Supplementary Table 3). **B:** Immunoblot analysis confirming the ability of erdafitinib in abrogating intracellular signaling in FGFR3::TACC3 WT Ba/F3 cells, while resistance was observed in N540K, V553M, V555L, V555M and L608V mutants. **C:** Higher concentrations of pemigatinib (100-300 nM) were required to abrogate FGFR3 signaling in FGFR3::TACC3 V553L Ba/F3 compared to erdafitinib (10-30 nM). **D:** Exposing FGFR3::TACC3 WT, N540K and V555L Ba/F3 cells corroborated findings of resistance to erdafinitib, and confirmed the role of “molecular brake disruption” of N540K mutant. **E-F:** Functional characterization of FGFR2 L551F mutation in Ba/F3 cells harboring *FGFR2::BICCl* transcript. A representative study of cell viability assay is reported on the top (**E**), and IC50 values (nM) are reported on the left as means of ≥ 3 independent data sets (**F**). WT: Wild-type.

In order to evaluate the functional impact of FGFR3 kinase domain mutations on the activity of FGFR inhibitors, we generated Ba/F3 cells with *FGFR3::TACC3* fusion harboring either N540K, V553L, V553M, V555L, V555M or L608V mutations. The expression levels of FGFR3::TACC3 exogenous fusion protein were homogenous across cell lines (Supplementary Figure 4). Ba/F3 cells were exposed to increasing doses of the FGFR inhibitors erdafitinib, infigratinib, pemigatinib, rogaratinib, futibatinib, derazantinib, AZD4547 and zoligratinib, to determine their corresponding IC50 values (Figure 4A and Supplementary Table 3). LY2874455 had an IC50 of 87.5 nM against parental Ba/F3 cells (without *FGFR3::TACC3* fusion), suggesting the drug exerted off- target effects on proliferation. In light of this observation, and considering that LY2874455 is no longer in clinical development, we did not include it in the pharmacological experiments.

The 8 selective FGFR inhibitors had IC50 values in the sub-nanomolar/nanomolar range against *FGFR3::TACC3* wild-type (WT) driven Ba/F3 cell lines. Each FGFR3 kinase domain mutation conferred a variable increase in the IC50 values to FGFR inhibitors. The IC50 against the frequent N540K mutant were > 100 nM for all the agents with the exception of erdafitinib and futibatinib (5 times lower). Similarly, erdafitinib and futibatinib retained low activity against the gatekeeper V555L/V555M, V553M and L608V mutations. The V553L mutant appeared to confer a moderate degree of resistance to FGFR inhibitors, as IC50 values were close to 10 nM for five inhibitors, but at sub-nanomolar/nanomolar concentrations for erdafitinib, infigratinib and futibatinib. Of note, this mutation emerged in a patient progressing on pemigatinib (MR406, Figure 3A, 3D).

To corroborate our observations, we performed immunoblot analyses with increasing concentrations of erdafitinib in Ba/F3 cells harboring a WT or a mutated *FGFR3::TACC3* construct, in presence of stimulation with heparin and acidic FGF (aFGF). Analysis of the intracellular signaling across models confirmed the viability assays. FGFR3, AKT and ERK phosphorylation were abrogated after treatment with low concentrations of erdafitinib in the WT model, while higher doses were required in N540K, V553M, V555L and L608V mutants (Figure 4B). FGFR3 phosphorylation and intracellular signaling were maintained in the presence of high doses of FGFR inhibitors with the V555M mutation. We confirmed that, compared with pemigatinib, lower concentrations of erdafitinib were sufficient to abrogate intracellular signaling in the V553L mutant (Figure 4C). This sustains the hypothesis of an efficient sequential treatment with erdafitinib in case of the emergence of a V553L mutation, which would also be the case with infigratinib and futibatinib according to the viability assays (Figure 4A).

When Ba/F3 cells having FGFR3::TACC3 WT, FGFR3::TACC3 N540K and FGFR3::TACC3 V555L were exposed to 30 nM of erdafitinib, the inhibitor was able to abolish the signaling only in the WT model (Figure 4D). Of note, in the untreated condition, we noticed an increased signaling in both mutants, which was especially marked in the N540K mutant in terms of basal ERK phosphorylation, in line with a “molecular brake” nature of the N540K mutation (15,16).

We also aimed to validate the role of FGFR2 L551F mutation in conferring resistance to pemigatinib in patient MR904, and to seek for alternative agents retaining activity against the mutant. We generated Ba/F3 cells harboring *FGFR2::BICC1* with the L551F mutation, and exposed them to the 8 FGFR inhibitors (Figure 4E, F). In addition to confirming pemigatinib resistance induced by the L551F mutation, our data revealed that erdafitinib and futibatinib could overcome this mutation.

#### Combining FGFR and PIK3CA inhibition is synergic in a resistant model of FGFR-driven urothelial cancer harboring an activating PIK3CA mutation

**Figure 5:**
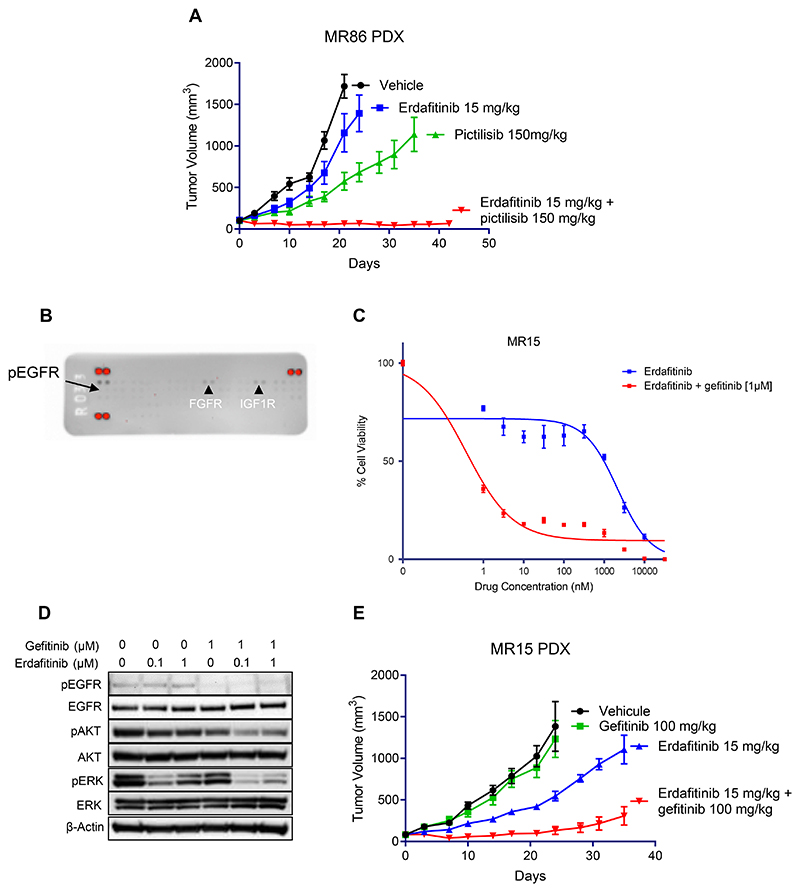
Synergistic effect of combinatorial treatments in patient-derived models of *FGFR*-driven urothelial cancer. **A:** In MR86 patient-derived xenograft (PDX), combined erdafitinib/pictilisib (PI3K inhibitor) administration had synergistic effect leading to prolonged inhibition of tumor growth. **B:** Protein extracts from MR15 cell line subjected to phospho-receptor tyrosine kinase (RTK) array revealed an EGFR activation. **C-D:** Concomitant EGFR and FGFR inhibition with gefitinib and erdafitinib was synergic both in viability assays (**C**) and in immunoblot analyses (**D**). **E:** The *in vivo* pharmacologic experiment in the corresponding PDX confirmed the efficiency of the combined treatment strategy.

To elucidate the potential role of PIK3CA E545K in resistance to FGFR inhibitors, we established patient-derived models as previously described (17). In the corresponding MR86 patient-derived xenograft (PDX) model, we evaluated the tumor growth using erdafitinib and pictilisib (PI3K inhibitor) as single agents or in combination. As depicted in Figure 5A, only the combination treatment was efficient in inhibiting the tumor growth suggesting that, while responses in patients with a PIK3CA preexisting mutation are possible, the PIK3CA E545K mutation contributes to the oncogenic signaling and its targeting seems relevant to maximize tumor response.

#### Non mutational resistance mechanism identified using patient-derived models

Patient MR15 suffered from an upper urothelial tumor driven by FGFR3 S249C. At progression to erdafitinib, the tissue biopsy did not reveal any additional genomic alterations compared with the baseline sample. A PDX and patient-derived cell line were established. After confirming that the 8 FGFR inhibitors could not restore sensitivity in the patient-derived cell line, we performed a phospho-receptor tyrosine kinase (RTK) array and detected hyperphosphorylation of EGFR (Figure 5B). While erdafitinib and the EGFR inhibitor gefitinib did not have a significant effect on cell growth inhibition as single-agents, the combinatorial treatment showed a synergistic effect in the MR15 cell line (Figure 5C and Supplementary Figure 5). Immunoblot analyses confirmed that the double inhibition was required to inhibit intracellular signaling (Figure 5D). The *in vivo* pharmacological study confirmed the synergistic effect of combining erdafitinib and gefitinib (not active as single agents) in inhibiting tumor growth in MR15 PDX (Figure 5E).

## Discussion

The positive clinical data of FGFR inhibitors in *FGFR*-driven bladder cancer provided the first proof-of-concept for precision medicine in urothelial cancer. Nevertheless, the ORR of 40% and the median PFS of 5.5 months observed with erdafitinib indicate that there is space for improvement in the field of FGFR targeted therapies for urothelial malignancies (10). Understanding the mechanisms of resistance to FGFR inhibitors represent a key step in developing new treatment strategies to improve the overall response and the durability of clinical benefit for patients in the setting of molecular-guided therapies.

In this study, we identified both on-target and off-target mechanisms of resistance to FGFR inhibitors in *FGFR*-driven urothelial carcinoma. Our data sustain the complementarity of the information derived from both tissue biopsies and ctDNA. If ctDNA is able to catch the heterogeneity of molecular modifications arising across tumor lesions, tissue biopsy is informative in the case of low ctDNA shedding, especially when disease progression is limited to the thorax, with mild tumor burden (18,19). In addition, tissue biopsy allows the establishment of patient- derived models, which are essential tools to further understand resistance and to test new treatment and combination strategies (20). We were not able to identify the resistance mechanism in 5 out of 21 patients (24%) included in our cohort. While this proportion of “unknown resistance mechanisms” can be considered as low, likely thanks to the availability of multiple sources of molecular analysis at progression to FGFR inhibitors (blood, tissue, patient-derived models), it remains challenging to systematically characterize the cause of resistance when no explanatory mutational event is detected, or when tumor-cell-extrinsic factors within the tumor microenvironment contribute to the resistance.

Across our cohort of 19 *FGFR3*-driven urothelial cancers, we identified FGFR3 kinase domain mutations in 7 patients at progression to FGFR inhibitors (37%). Of note, the characteristics of the FGFR3 kinase domain at resistance are distinctly different from what has been observed in *FGFR2*-driven cholangiocarcinoma. In patients suffering from cholangiocarcinoma, the occurrence of polyclonal *(i.e.* multiple) FGFR2 kinase domain mutations is frequently reported at progression to reversible FGFR inhibitors (21–23). In our study, multiple FGFR3 kinase domain mutations were detected only in patient CTC1845 (N540K, V555L, L608V). The sole availability of ctDNA (lack of tissue biopsy) at the moment of progression to futibatinib in this patient previously treated with erdafitinib, precludes the assessment of the potential serial acquisition of the mutations, and their allelic status could not be determined due to ctDNA amplicon size limitation. Pal *et al.* have previously reported the detection of V555L/M and L608V in 4 patients at progression to erdafitinib, but only one case had a polyclonal presentation (V555M and L608V) (11). In addition to these 2 mutations, we reported for the first time additional mutations occurring in the FGFR3 kinase domain mutation at progression to selective inhibitors: N540K, V553L, V553M and E587Q. With the exception of the latter, all the other mutational events have been reported in their corresponding positions of FGFR2 (N550K, V563L, V565I/L/F, L618V, according to the NM-001144913.1 transcript), suggesting that these kinase domain conserved residues are resistance hotspots in the FGFR3 kinase domain as well (21–23). Whereas it is difficult to derive the incidence of precise mutations from our study with a limited sample size, the gatekeeper residue FGFR3 V555 and the molecular brake N540 could represent relevant targets to consider for the development of novel inhibitors due to their higher frequency and resistance pattern.

Although confirming the role of FGFR3 kinase domain mutations in conferring resistance to the inhibitors, our functional studies were not informative in recognizing agents able to overcome on-target resistance. The only exception was represented by the potential role of erdafitinib, infigratinib and futibatinib against the V553L mutation acquired in the post-pemigatinib sample. Of note, among reversible FGFR inhibitors, erdafitinib yielded the lowest IC50 values against both WT and mutated *FGFR3:TACC3* Ba/F3 cell lines, potentially explaining its better outcomes observed in clinical trials.

The immunoblots and cell viability analyses highlighted the differential contribution of molecular brake (N540K) and gatekeeper (V555L) mutation in conferring resistance. V555L, thought to interfere with drug binding (24), boosts intracellular signaling as well, mainly represented by ERK phosphorylation. This latter was even more marked in presence of N540K, in line with the “molecular brake” function of the N540 residue (15,16). From this point of view, novel inhibitors will need to harbor higher potency, and their design should allow them to circumvent the steric clash induced by gatekeeper (and other) mutations.

The functional evidence generated regarding FGFR2 L551F mutation also suggested it could be therapeutically targeted in *FGFR2*-driven tumors. In our patient MR904 the mutation emerged at progression to pemigatinib, and Varghese *et al* reported a similar finding at progression to infigratinib in a patient affected by an *FGFR2*-driven cholangiocarcinoma (one additional patient developed L551F in the context of polyclonal FGFR kinase domain mutations) (21). FGFR2 L551F mutation was not previously characterized, and our data suggest that L551F, while engendering resistance to pemigatinib and infigratinib, could be overcome by futibatinib and erdafitinib.

In addition to on-target alterations responsible for resistance, we identified recurrent events affecting the PI3K/mTOR pathway at resistance to FGFR inhibitors in urothelial tumors, concomitantly or not with FGFR3 kinase domain mutations. In our population and in molecular landscape studies of urothelial tumors, *PIK3CA* and *TSC1/2* alterations are not mutually exclusive with *FGFR3* in baseline samples (1–5,25). Nevertheless, their enrichment in post-progression samples strongly implicates their involvement in resistance. As supported by models derived from patient MR86, a baseline activating mutation in *PIK3CA* may not be fully capable of conferring resistance, however may represent a suitable target to improve responses. The observed alterations in the PI3K/mTOR pathway in clinical samples from this study have been shown to be responsible for progression to targeted therapies in other oncogene-driven disease and suitable targets for specific inhibition (26–31).

The establishment of the patient-derived cell line and PDX of patient MR15 illustrates the relevance of such models in providing critical elements in addressing off-target, non-genomic mechanisms of resistance and enables the development of new strategies for treatment paradigms. Of note, EGFR bypass activation has been reported as an early event in the setting of FGFR inhibition in *FGFR3*-altered urothelial cancer and in *FGFR2*-driven cholangiocarcinoma, suggesting the opportunity of EGFR targeting in progressive *FGFR*-driven diseases (32,33).

This study has several limitations. While being the largest study on the subject thus far, the limited sample population precludes the generalization of our findings. Despite the availability for the majority of the patients, pre-treatment sequencing analyses could not be performed systematically. The lack of a matched collection of post-progression tissue samples and ctDNA in some patients limits the depth of the molecular insights we could obtain. Despite our systematic attempt in establishing patient-derived models, the relatively low success rate limited the identification of non- genetic driven resistance mechanisms. Finally, we did not validate functionally the role of *TSC1/2* mutations neither their allelic status.

Altogether, our study represents the first systematic approach towards understanding resistance to FGFR inhibitors in *FGFR*-driven urothelial cancer. The evidences generated on both on-target and off-target molecular events responsible for resistance will serve as the backbone to address the development of new therapeutic strategies to improve patients’ outcomes.

## Methods

### Patients and treatments

To be included in this study, patients had to satisfy the following criteria: 1) diagnosis of an advanced urothelial cancer; 2) treatment with a selective FGFR inhibitor due to the detection of a molecular alteration in one of the *FGFR* family members; 3) availability of post-progression tissue and/or ctDNA available for molecular analyses.

Patients started FGFR inhibitor treatment between 2013 and 2021. Patients were treated in the setting of clinical trials or compassionate use programs allowing treatment with FGFR inhibitors on the basis of molecular selection. Disease response was measured according to RECIST, and PFS was calculated from the date of targeted inhibitor start to the day of radiological evidence of progression.

The molecular analyses were performed within institutional studies ongoing at Gustave Roussy whose aim is the molecular characterization of tumors: MATCH-R (NCT02517892) (17), MOSCATO (NCT01566019) (34), STING-UNLOCK (NCT04932525) and CTC (NCT02666612).

All patients participating in the mentioned studies were fully informed and signed a written informed consent. The studies have been approved by the ethics committee at Institut Gustave Roussy, the French National Agency for Medicines and Health Products Safety (ANSM), and are conducted in accordance with the Declaration of Helsinki.

The objectives of MOSCATO and MATCH-R studies are to address patients to targeted treatments according to molecular alterations, and to assess molecular mechanisms of resistance, respectively. These studies initially focused on molecular analysis performed on tissue biopsy, when ctDNA technologies were not commonly used and blood/plasma samples not collected (see MOSCATO patient M521, and MATCH-R MR15 and MR86). On the other hand, CTC and STING-UNLOCK were developed to study molecular alterations from blood samples (exclusively for CTC, including tissue biopsy for STING-UNLOCK). The availability of post-progression samples relied on the precise study in which individual patients were enrolled.

### Molecular analyses

Post-progression tumor, when possible, underwent WES, with or without concomitant RNAseq. The main limitation for WES/RNAseq performance was the proportion of tumor cells ≥ 30% in the tissue sample. In cases where the proportion of tumor cells was between 10% and 30%, molecular analyses with targeted NGS panels (Mosc-3, Oncomine v3) were performed. For WES, the mean coverage was 140X.

ctDNA analyses were performed with Illumina or Foundation Medicine liquid biopsy panels. Importantly, all ctDNA sequencing had a complete coverage of the kinase domains of *FGFR* genes. Briefly, extracted cfDNA from patient samples were processed through a library preparation that includes addition of unique molecular identifiers followed by a hybrid capture based workflow and high-depth sequencing. For each patient with longitudinal ctDNA assessment, only analyses performed with the same platform were reported.

### Site-directed mutagenesis

Lentiviral vectors expressing *FGFR3:TACC3* fusions were created using the pLenti6/V5 directional TOPO Cloning Kit (#K495510, Thermo Fisher Scientific) according to the manufacturer’s instructions. Point mutations in the FGFR3 kinase domain of the *FGFR3:TACC3* fusion were introduced using the QuickChange XL Site-Directed Mutagenesis Kit (#200516, Agilent) according to manufacturer's protocol using the following primers:

FGFR3 N540K forward (F) CAAAAACATCATCAAGCTGCTGGGCGCCTGC
FGFR3 N540K reverse (R) GCAGGCGCCCAGCAGCTTGATGATGTTTTTG
FGFR3 V553L F CGCAGGGCGGGCCCCTGTACTTGCTGGTGGAGTACGCGGCC
FGFR3 V553L R GGCCGCGTACTCCACCAGCAAGTACAGGGGCCCGCCCTGCG
FGFR3 V553M F CGCAGGGCGGGCCCCTGTACATGCTGGTGGAGTACGCGGCC
FGFR3 V553M R GGCCGCGTACTCCACCAGCATGTACAGGGGCCCGCCCTGCG
FGFR3 V555L F GCCCCTGTACGTGCTGCTGGAGTACGCGGCCAA
FGFR3 V555L R TTGGCCGCGTACTCCAGCAGCACGTACAGGGGC
FGFR3 V555M F GCCCCTGTACGTGCTGATGGAGTACGCGGCCAA
FGFR3 V555M R TTGGCCGCGTACTCCATCAGCACGTACAGGGGC
FGFR3 L608V F AGGTGGCCCGGGGCATGGAGTACGTGGCCTCCCAGAAGTGCATCCAC
FGFR3 L608V R GTGGATGCACTTCTGGGAGGCCACGTACTCCATGCCCCGGGCCACCT

The same procedure was used to create lentiviral vectors expressing *FGFR2::BICC1*, and the primers used for creating L551F mutations were as follows:

FGFR2 L551F F GGGAAACACAAGAATATCATAAATTTTCTTGGAGCCTGCACACAGGATG
FGFR2 L551F R CATCCTGTGTGCAGGCTCCAAGAAAATTTATGATATTCTTGTGTTTCCC

### Allelic distribution of FGFR3 mutations

To assess the allelic status of FGFR3 S249C and V553L in patient MR406, mRNA obtained from the tissue biopsy underwent retro-transcription, and a PCR spanning the two mutated residue was performed on cDNA. Amplicons were subcloned into pCR2.1-TOPO vector (Invitrogen) according to the manufacturer’s protocol. Individual cDNA was sequenced by Sanger sequencing to determine the cis/trans status of mutations.

### Development of patient-derived xenografts in mice and in vivo pharmacologic studies

All animal procedures and studies have been approved by the French Ministry of ‘Education nationale, de l’Enseignement supérieur et de la Recherche’ (APAFIS#279O-2015112015055793 and APAFIS#2328-2015101914074846). Fresh tumor fragments were implanted in the subrenal capsule of 6-week-old female NOD scid gamma (NSG) mice obtained from Charles River Laboratories. Patient-derived xenograft (PDX)-bearing NSG mice were treated with gefitinib (100mg/kg qd in 0.5% methylcellose, 0.5% tween80) or erdafitinib (15 mg/kg qd in 10% HP-beta-CD (hydroxypropyl beta cyclodextrin) or pictilisib (150mg/kg qd in 0.5% methylcellose, 0.5% tween80) alone or in combination by oral gavage. Eight mice per group were treated for up to 40 days and tumor volume and mice weight were measured twice per week.

### Cell lines

Parental Ba/F3 cells were purchased from DSMZ (ref. ACC 300) and cultured in DMEM 10% FBS in the presence of IL-3 (0.5 ng/mL). Ba/F3 cells were infected with lentiviral constructs, as reported previously (35), to express the *FGFR3::TACC3* or the *FGFR2::BICC1* fusions, with a WT kinase domain or with the mentioned kinase domain mutations. Infected Ba/F3 cells harboring were selected in the presence of blasticidin (14 mg/mL) and IL-3 (0.5 ng/mL) until recovery, and a second selection by culturing the cells in the absence of IL-3. *FGFR2*/3 fusions and corresponding kinase domain mutations were confirmed on the established cell lines by Sanger sequencing.

After several attempts, we were not able to establish IL-3 independent Ba/F3 cells driven by FGFR3 S249C or FGFR3 Y373C mutations. Previous experience showed that FGFR2 cannot drive IL-3 independent proliferation in the same way than FGFR1 (16), and we envisage that FGFR3 mutations occurring in the extracellular domain, oncogenic drivers in human tumors, are not sufficient to fulfill this function in Ba/F3 models in isolation.

Patient-derived cell lines (MR15) were developed from patient-derived xenograft (PDX) samples by enzymatic digestion with a tumor dissociation kit (ref. 130- 095-929, Miltenyi Biotec) and mechanical degradation with the GentleMACs dissociator. Cells were cultured with DMEM/F-12 þ GlutamMAX 10% FBS and 10% enriched with hydrocortisone 0.4 mg/mL, cholera toxin 8.4 ng/mL, adenine 24 mg/mL, and ROCK inhibitor 5 mmol/L (Y-27632, S1049 Selleckchem) until a stable proliferation of tumor cells was observed, as described previously (36). Culture media was then transitioned to DMEM and cultured in the presence of erdafitinib 300 nmol/L to 1 mmol/L.

As for cell verification, we confirmed that engineered Ba/F3 as well as MR15 patient-derived cell line harbored the corresponding FGFR fusion and mutations by RT-PCR and Sanger sequencing. The cells were not tested for mycoplasma contamination but cells were not maintained in culture for more than 2 months in culture after establishment or thawing.

### Reagents

Erdafitinib, infigratinib, pemigatinib, futibatinib, derazantinib, AZD4547, zoligratinib and LY2874455 were purchased from Selleck Chemicals. Rogaratinib was purchased from MedChemExpress.

### Cell viability assays and immunoblots

Cell viability assays were performed in 96-well plates using the CellTiter Glo Luminescent Cell Viability Assay (G7570, Promega). For Ba/F3, we seeded 4000 cells/well and we treated cells for 48h. For MR15 patient-derived cell line, we seeded 3000 cells/well and we treated cells for five days.

Ba/F3 cells with *FGFR3::TACC3* fusion with a WT or a mutated kinase domain were treated for 24h with the corresponding doses of FGFR inhibitors in presence of Heparin 25 ug/ml (Sigma Aldrich, ref H3149) and aFGF 50 ng/ml (R&D systems, ref 232-FA-025/CF), as previously reported (37).

For Western blot assays, the following antibodies were purchased from Cell Signaling Technology: pFGFR (3471S), pAKT (4060S), AKT (4691S), pERK (9101S), ERK (9102S), pS6 (4858S), S6 (2217S), BIM (2933S). Anti-FGFR3 (ab133644) and anti-pEGFR (ab5644) antibodies was purchased from Abcam; anti-EGFR (sc-03) antibody from SantaCruz and anti-β-actin (A1978) from Sigma Aldrich.

## Supplementary Material

Supplementary figure 1

Supplementary figure 2

Supplementary figure 3

Supplementary figure 4

Supplementary figure 5

Supplementary table 1

Supplementary table 2

Supplementary table 3

## Figures and Tables

**Table 1:** Off-target molecular alterations in the PI3K/mTOR pathway detected in patients suffering from *FGFR3-driven* urothelial cancers. Eleven out of 19 (58%) patients harbored alterations in genes belonging to the PI3K/mTOR pathway, with an enrichment in post-progression samples. Molecular alterations reported in bold were not present in baseline samples. ctDNA: Circulating tumor DNA; VAF: Variant allele frequency; NA: Not assessed; BOR: Best objective response; PFS: Progression-free survival; ampl: Amplification; CNV: copy number variation.

Patient	Baseline tissue analysis	Baseline ctDNA(VAF)	FGFR inhibitor	BOR	PFS(months)	Post-progression tissue analysis	Post-progression ctDNA analysis (VAF)
**MR15**	FGFR3 S249C PIK3CA E545A	NA	Erdafitinib	-30%	5.8	FGFR3 S249C PIK3CA E545A	NA
**MR86**	FGFR3 S249C PIK3CA E545K	NA	Erdafitinib	-25%	2.7	FGFR3 S249C **FGFR3 N540K** PIK3CA E545K	NA
**ST2267**	FGFR3::TACC3PIK3CA H1047R	FGFR3::TACC3 8.03%	Futibatinib	+24%	1.7	NA	FGFR3::TACC3 4.16%
**MR336**	FGFR3 S249C	FGFR3 S249C 7.72%	Erdafitinib	-22%	5.6	FGFR3 S249C **PIK3CA E545K**	FGFR3 S249C 10.38% **PIK3CA E545K 7%**
**MR410**	FGFR3 S249C TSC1 S561fs	NA	Erdafitinib	-46%	19.6	FGFR3 S249C FGFR3 ampl (>10 copies)TSC1 S561fs **PIK3CA E726K**	FGFR3 S249C 45.94% **FGFR3 E589Q 31.12%** TSC1 S561fs 69.96%
**M322**	FGFR3::TACC3	FGFR3::TACC3 0.65%	Erdafitinib	-40%	3.7	**TSC2 P28fs**	**FGFR3:TACC3 2.95% FGFR3 V555M 0.37%** **TSC2 P28fs 0.34%**
**M521**	FGFR3::TACC3	NA	Erdafitinib	-39%	8.3	FGFR3-TACC3 TSC1 Q865*	NA
**MR1105**	FGFR3::TACC3	NA	Futibatinib	-35%	7.7	NA	FGFR3::TACC3 9.8% **TSC1 A186fs 1.8%**
**ST701**	NA	FGFR3 S249C 0.2%	Erdafitinib	-39%	4.1	TSC1 Q830*	FGFR3 S249C 0.45% TSC1 Q830* 1.8%
**MR246**	FGFR3 S249C	NA	Erdafitinib	-21%	3.4	FGFR3 S249C	FGFR3 S249C 2.37% **NF2 L163fs 0.21% FGFR2 V517M 0.12%**
**M2057**	FGFR3 S249C	NA	Erdafitinib	+174%	1.4	**PTEN C136fs FGFR3 CNV (6 copies)**	FGFR3 S249C 48.13%

## Data Availability

WES/RNAseq raw data files from this study are deposited at the European Genome–Phenome Archive (EGA) using the accession code EGAS00001007335. Access to this shared dataset is controlled by the institutional Data Access Committee, and requests for access can be sent to the corresponding author. Further information about EGA can be found on https://ega-archive.org/. Any additional information required to reanalyze the data reported in this paper is available upon request from the corresponding author.

## Statement of significance

In the largest study on the topic thus far, we detected a high frequency of FGFR kinase domain mutations responsible for resistance to FGFR inhibitors in urothelial cancer. Off-target resistance mechanisms mainly involved the PI3K-mTOR pathway. Our findings provide preclinical evidences sustaining combinatorial treatment strategies to overcome bypass resistance.
