# Supplementary material for: Resistance to selective FGFR inhibitors in FGFR-driven urothelial cancer
Source: Cancer Discov. Author manuscript; Available in PMC 2023 Sep 7. (PMC10481128; doi:10.1158/2159-8290.CD-22-1441)
Supplement: Supplementary figure 1 [file EMS178531-supplement-Supplementary_figure_1.pptx]

## Slide 1
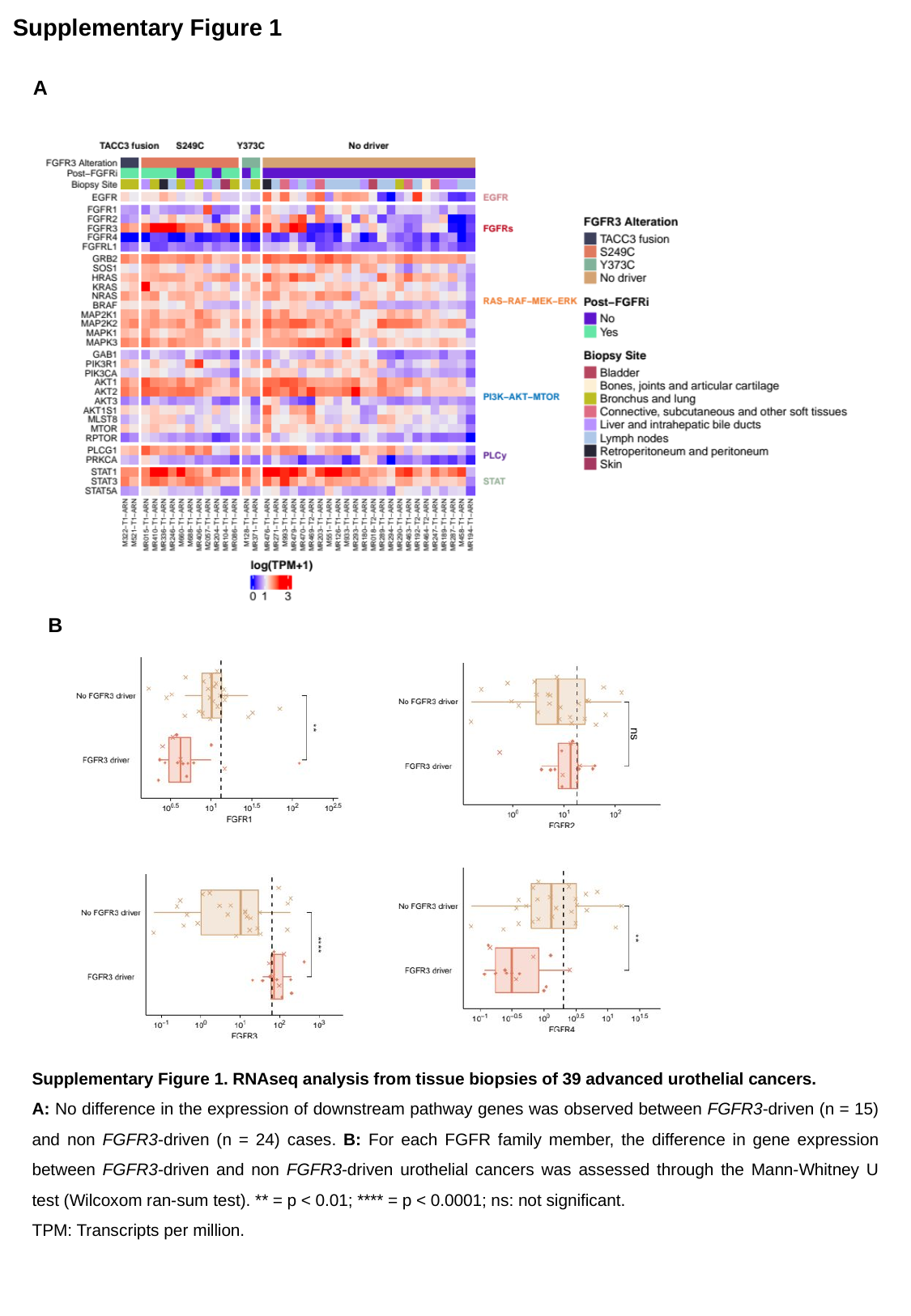

Supplementary Figure 1
A
 B
Supplementary Figure 1. RNAseq analysis from tissue biopsies of 39 advanced urothelial cancers.
A: No difference in the expression of downstream pathway genes was observed between FGFR3-driven (n = 15) and non FGFR3-driven (n = 24) cases. B: For each FGFR family member, the difference in gene expression between FGFR3-driven and non FGFR3-driven urothelial cancers was assessed through the Mann-Whitney U test (Wilcoxom ran-sum test). ** = p < 0.01; **** = p < 0.0001; ns: not significant.
TPM: Transcripts per million.
