# Supplementary material for: Resistance to selective FGFR inhibitors in FGFR-driven urothelial cancer
Source: Cancer Discov. Author manuscript; Available in PMC 2023 Sep 7. (PMC10481128; doi:10.1158/2159-8290.CD-22-1441)
Supplement: Supplementary figure 2 [file EMS178531-supplement-Supplementary_figure_2.pptx]

## Slide 1
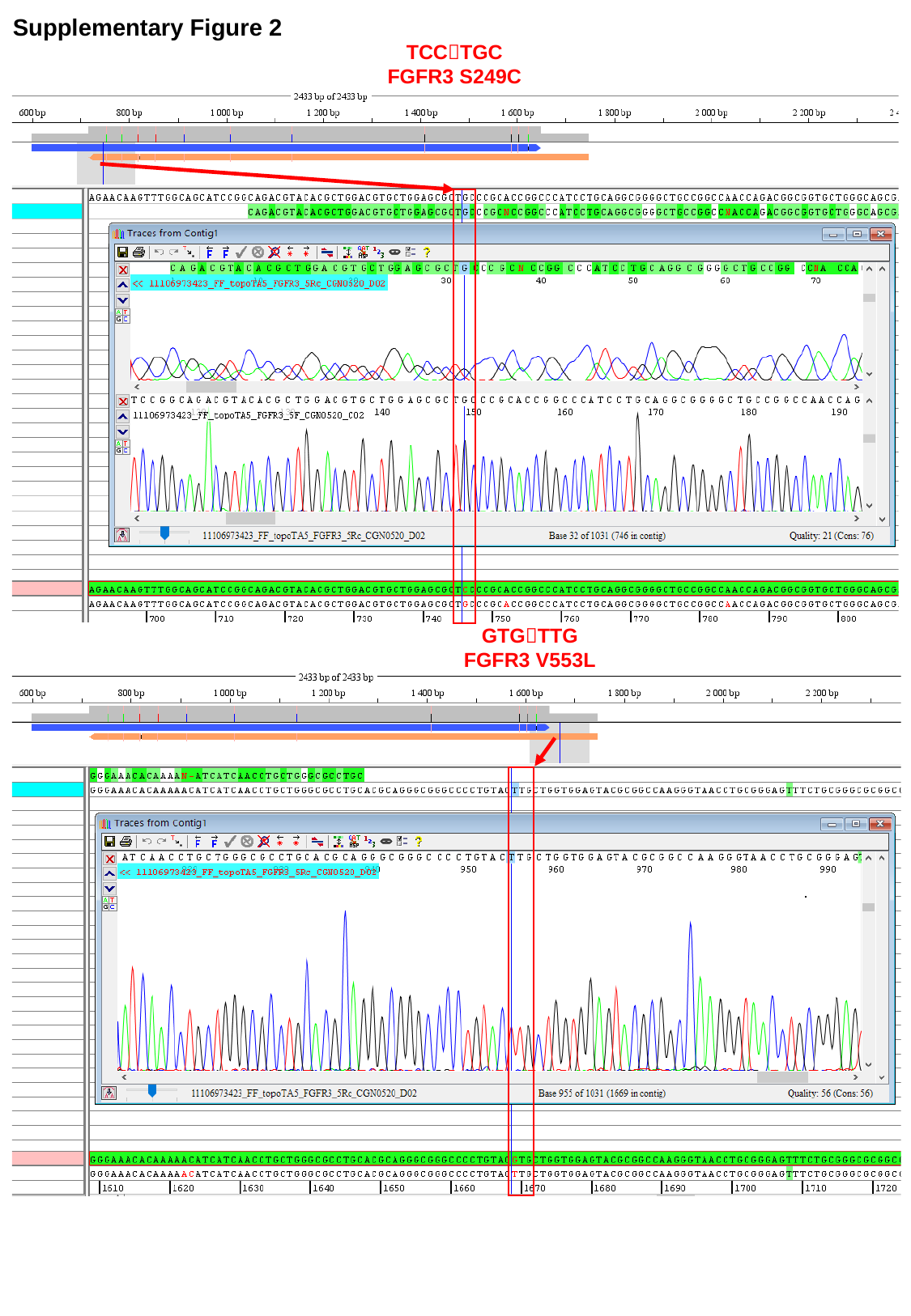

Supplementary Figure 2
TCCTGC
FGFR3 S249C
GTGTTG
FGFR3 V553L

## Slide 2
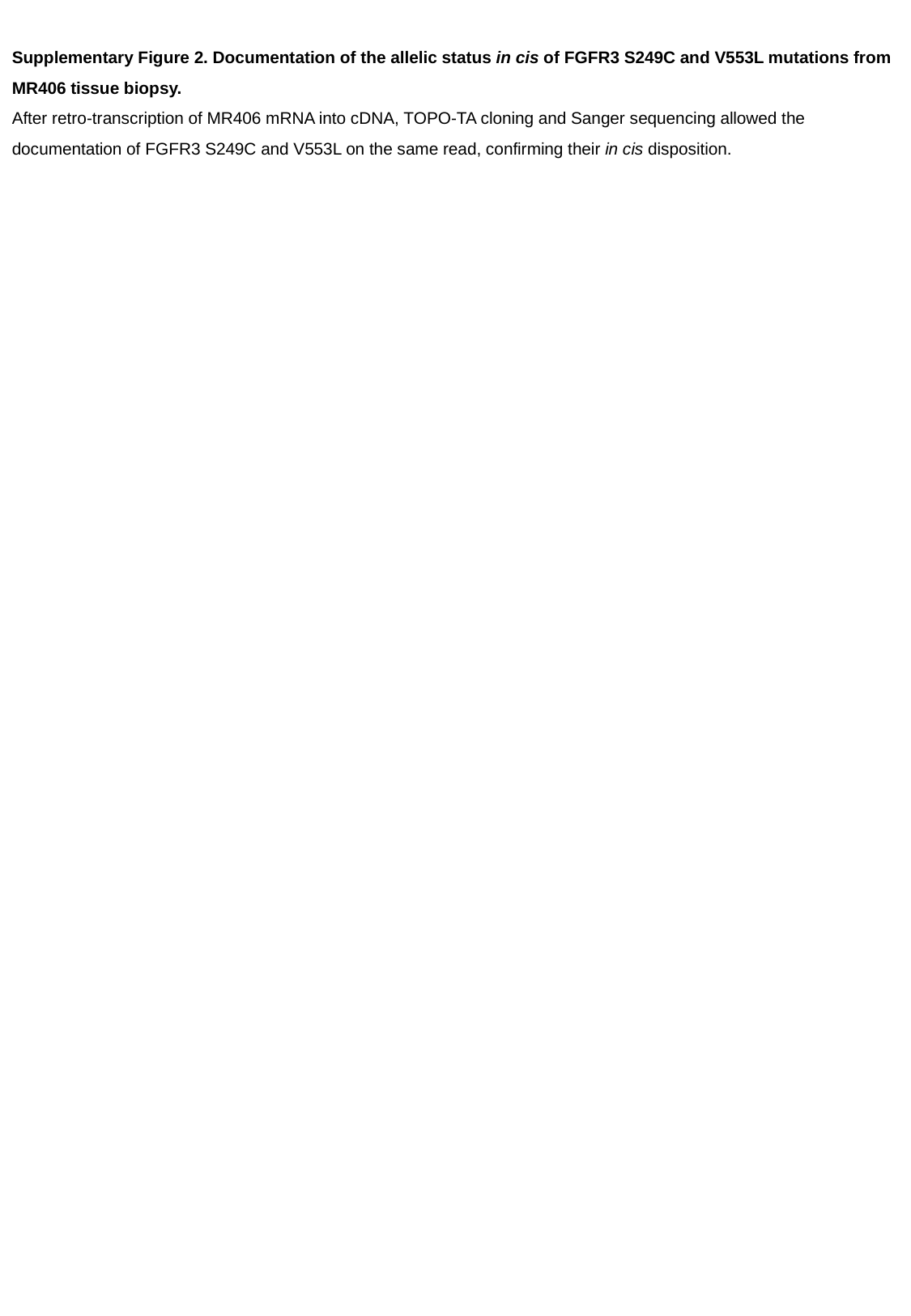

Supplementary Figure 2. Documentation of the allelic status in cis of FGFR3 S249C and V553L mutations from MR406 tissue biopsy.
After retro-transcription of MR406 mRNA into cDNA, TOPO-TA cloning and Sanger sequencing allowed the documentation of FGFR3 S249C and V553L on the same read, confirming their in cis disposition.
