# Supplementary material for: Resistance to selective FGFR inhibitors in FGFR-driven urothelial cancer
Source: Cancer Discov. Author manuscript; Available in PMC 2023 Sep 7. (PMC10481128; doi:10.1158/2159-8290.CD-22-1441)
Supplement: Supplementary figure 3 [file EMS178531-supplement-Supplementary_figure_3.pptx]

## Slide 1
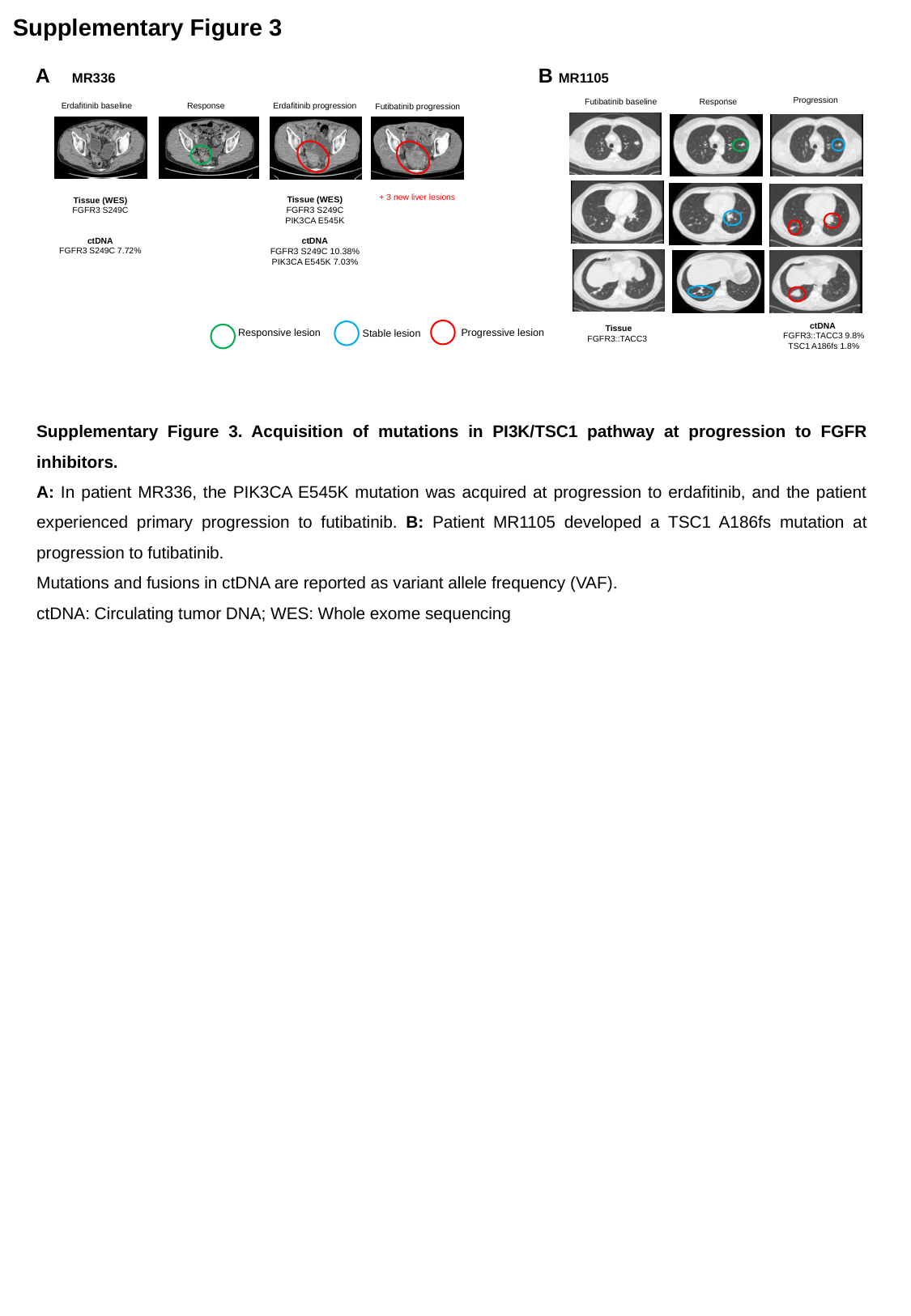

Supplementary Figure 3
B MR1105
 A MR336
Progression
Response
Futibatinib baseline
Erdafitinib baseline
Erdafitinib progression
Response
Futibatinib progression
+ 3 new liver lesions
Tissue (WES)
FGFR3 S249C
PIK3CA E545K
ctDNA
FGFR3 S249C 10.38%
PIK3CA E545K 7.03%
Tissue (WES)
FGFR3 S249C
ctDNA
FGFR3 S249C 7.72%
ctDNA
FGFR3::TACC3 9.8%
TSC1 A186fs 1.8%
Tissue
FGFR3::TACC3
Responsive lesion
Progressive lesion
Stable lesion
Supplementary Figure 3. Acquisition of mutations in PI3K/TSC1 pathway at progression to FGFR inhibitors.
A: In patient MR336, the PIK3CA E545K mutation was acquired at progression to erdafitinib, and the patient experienced primary progression to futibatinib. B: Patient MR1105 developed a TSC1 A186fs mutation at progression to futibatinib.
Mutations and fusions in ctDNA are reported as variant allele frequency (VAF).
ctDNA: Circulating tumor DNA; WES: Whole exome sequencing
