# Supplementary material for: Resistance to selective FGFR inhibitors in FGFR-driven urothelial cancer
Source: Cancer Discov. Author manuscript; Available in PMC 2023 Sep 7. (PMC10481128; doi:10.1158/2159-8290.CD-22-1441)
Supplement: Supplementary figure 4 [file EMS178531-supplement-Supplementary_figure_4.pptx]

## Slide 1
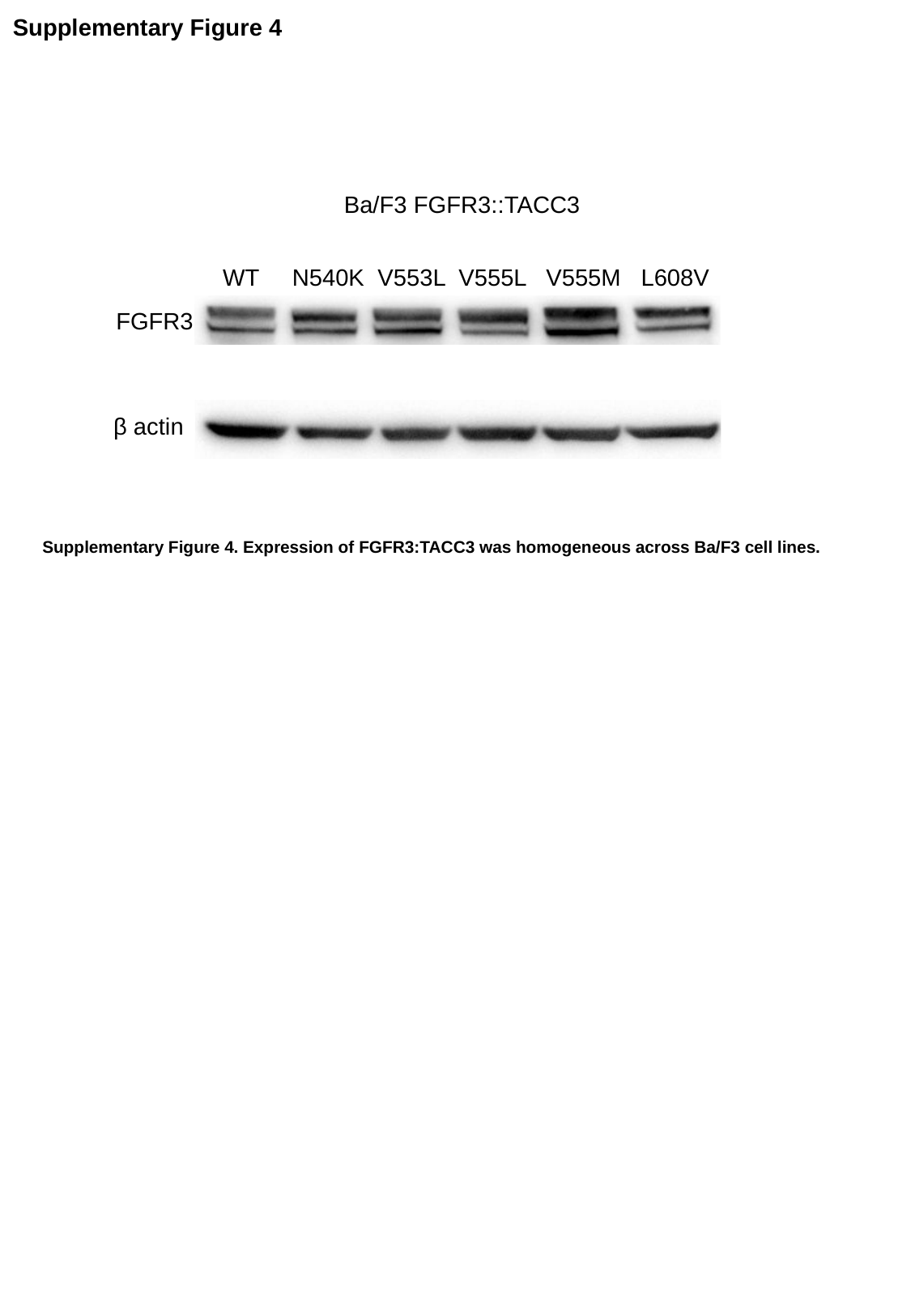

Supplementary Figure 4
Ba/F3 FGFR3::TACC3
 WT N540K V553L V555L V555M L608V
FGFR3
β actin
Supplementary Figure 4. Expression of FGFR3:TACC3 was homogeneous across Ba/F3 cell lines.
