# Supplementary material for: Resistance to selective FGFR inhibitors in FGFR-driven urothelial cancer
Source: Cancer Discov. Author manuscript; Available in PMC 2023 Sep 7. (PMC10481128; doi:10.1158/2159-8290.CD-22-1441)
Supplement: Supplementary figure 5 [file EMS178531-supplement-Supplementary_figure_5.pptx]

## Slide 1
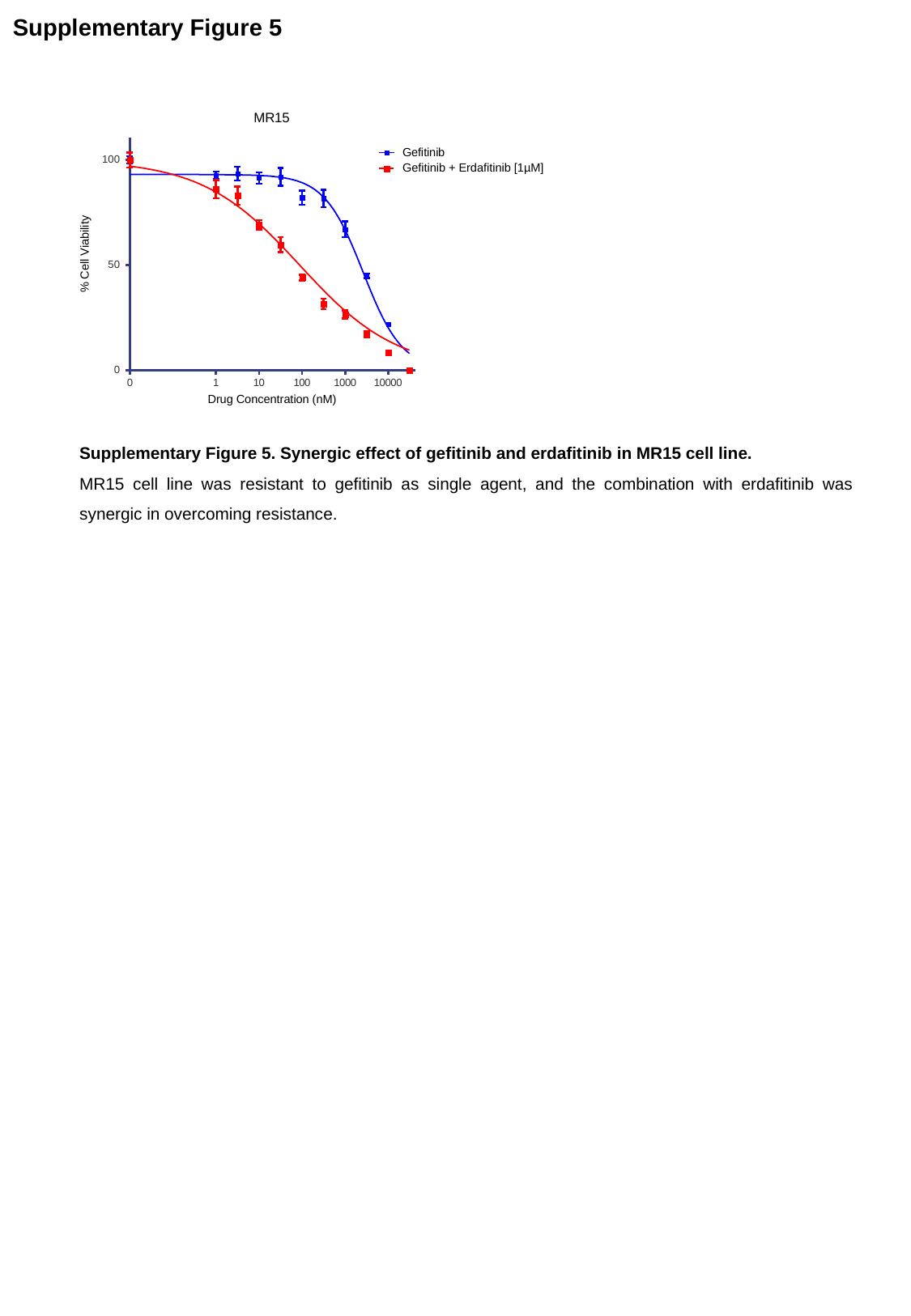

Supplementary Figure 5
Supplementary Figure 5. Synergic effect of gefitinib and erdafitinib in MR15 cell line.
MR15 cell line was resistant to gefitinib as single agent, and the combination with erdafitinib was synergic in overcoming resistance.
