# Supplementary material for: Resistance to selective FGFR inhibitors in FGFR-driven urothelial cancer
Source: Cancer Discov. Author manuscript; Available in PMC 2023 Sep 7. (PMC10481128; doi:10.1158/2159-8290.CD-22-1441)
Supplement: Supplementary table 1 [file EMS178531-supplement-Supplementary_table_1.pptx]

## Slide 1
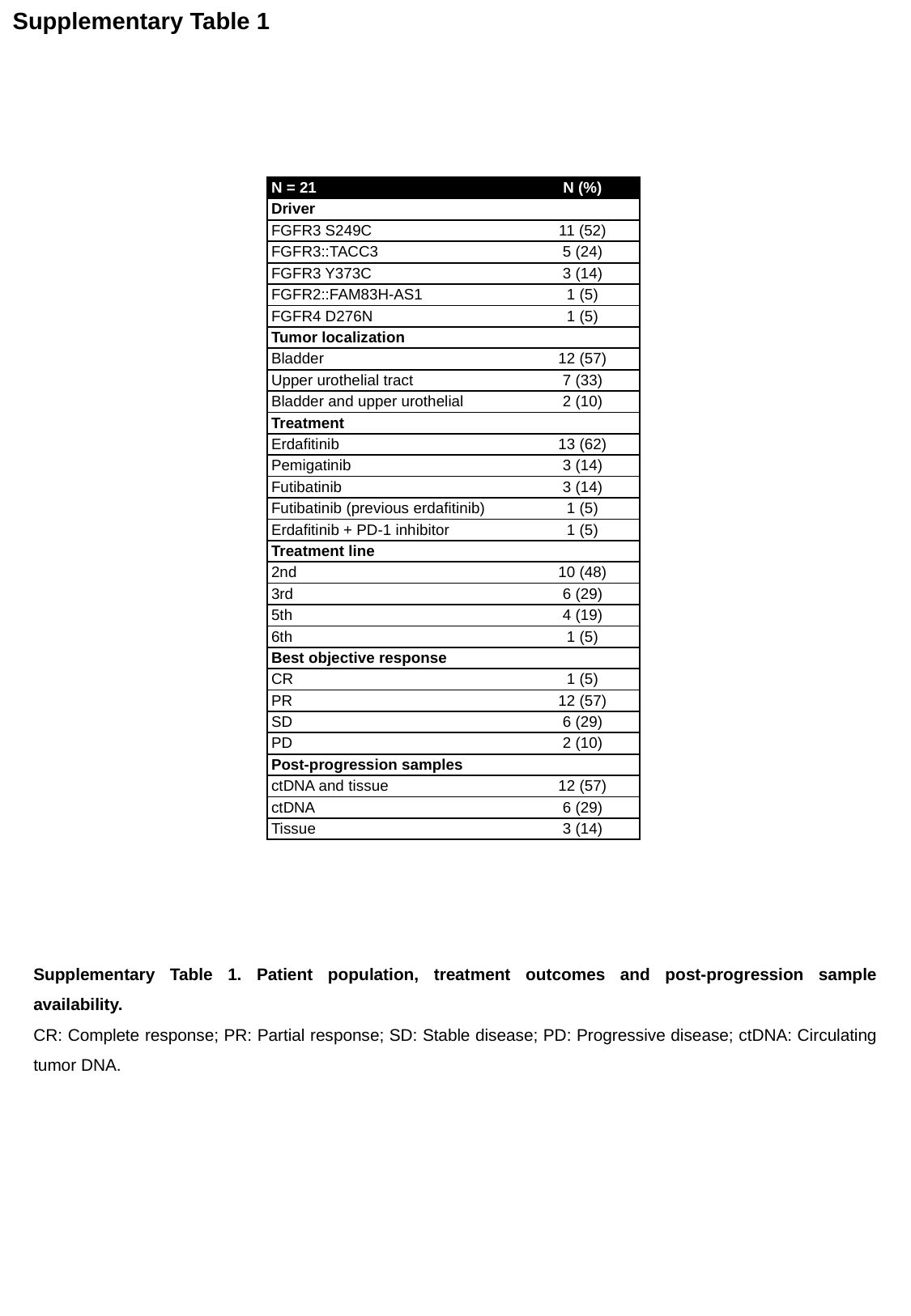

Supplementary Table 1
| N = 21 | N (%) |
| --- | --- |
| Driver | |
| FGFR3 S249C | 11 (52) |
| FGFR3::TACC3 | 5 (24) |
| FGFR3 Y373C | 3 (14) |
| FGFR2::FAM83H-AS1 | 1 (5) |
| FGFR4 D276N | 1 (5) |
| Tumor localization | |
| Bladder | 12 (57) |
| Upper urothelial tract | 7 (33) |
| Bladder and upper urothelial | 2 (10) |
| Treatment | |
| Erdafitinib | 13 (62) |
| Pemigatinib | 3 (14) |
| Futibatinib | 3 (14) |
| Futibatinib (previous erdafitinib) | 1 (5) |
| Erdafitinib + PD-1 inhibitor | 1 (5) |
| Treatment line | |
| 2nd | 10 (48) |
| 3rd | 6 (29) |
| 5th | 4 (19) |
| 6th | 1 (5) |
| Best objective response | |
| CR | 1 (5) |
| PR | 12 (57) |
| SD | 6 (29) |
| PD | 2 (10) |
| Post-progression samples | |
| ctDNA and tissue | 12 (57) |
| ctDNA | 6 (29) |
| Tissue | 3 (14) |
Supplementary Table 1. Patient population, treatment outcomes and post-progression sample availability.
CR: Complete response; PR: Partial response; SD: Stable disease; PD: Progressive disease; ctDNA: Circulating tumor DNA.
