# Supplementary material for: Resistance to selective FGFR inhibitors in FGFR-driven urothelial cancer
Source: Cancer Discov. Author manuscript; Available in PMC 2023 Sep 7. (PMC10481128; doi:10.1158/2159-8290.CD-22-1441)
Supplement: Supplementary table 2 [file EMS178531-supplement-Supplementary_table_2.pptx]

## Slide 1
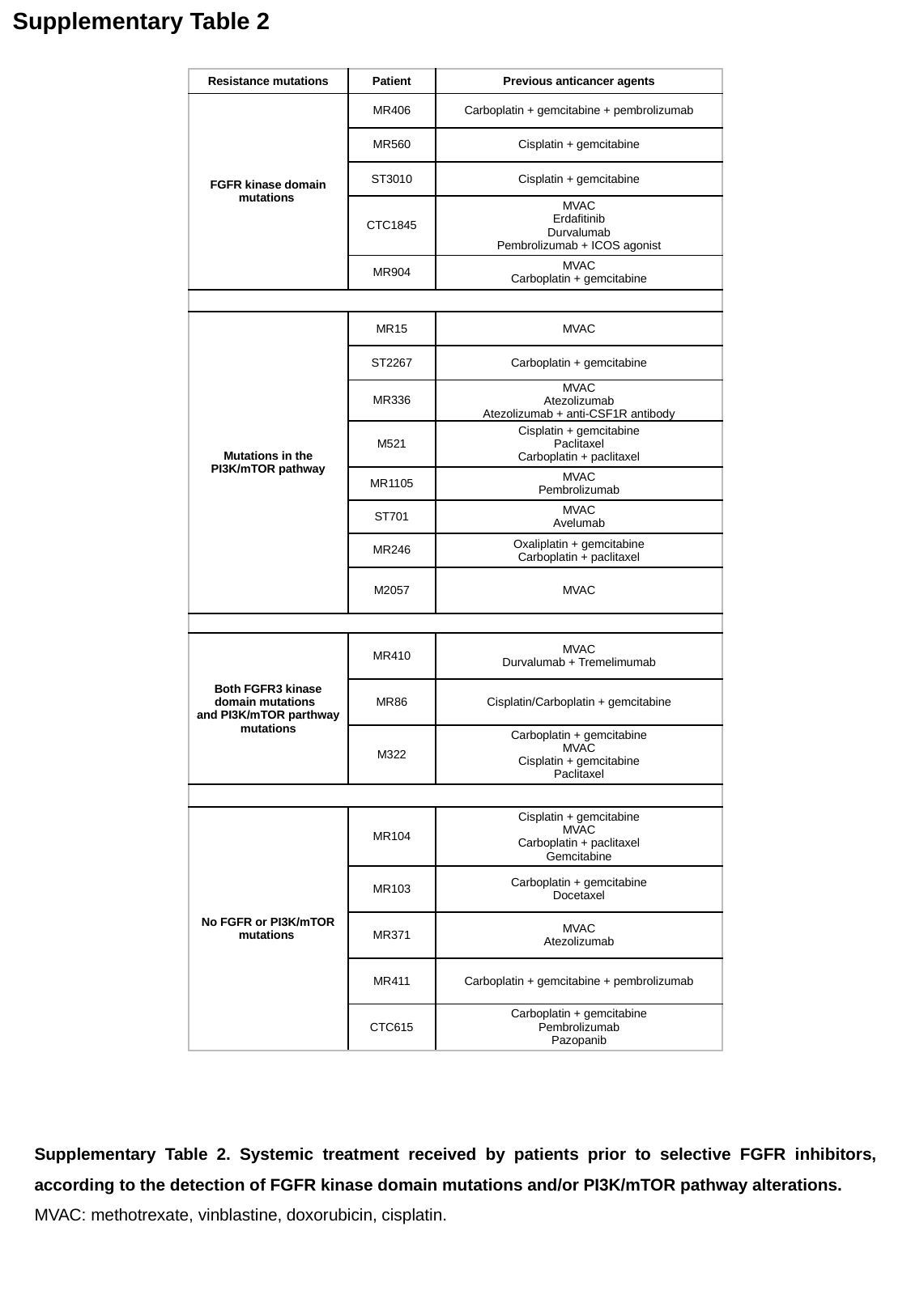

Supplementary Table 2
| Resistance mutations | Patient | Previous anticancer agents |
| --- | --- | --- |
| FGFR kinase domain mutations | MR406 | Carboplatin + gemcitabine + pembrolizumab |
| | MR560 | Cisplatin + gemcitabine |
| | ST3010 | Cisplatin + gemcitabine |
| | CTC1845 | MVAC Erdafitinib Durvalumab Pembrolizumab + ICOS agonist |
| | MR904 | MVAC Carboplatin + gemcitabine |
| | | |
| Mutations in the PI3K/mTOR pathway | MR15 | MVAC |
| | ST2267 | Carboplatin + gemcitabine |
| | MR336 | MVAC Atezolizumab Atezolizumab + anti-CSF1R antibody |
| | M521 | Cisplatin + gemcitabine Paclitaxel Carboplatin + paclitaxel |
| | MR1105 | MVAC Pembrolizumab |
| | ST701 | MVAC Avelumab |
| | MR246 | Oxaliplatin + gemcitabine Carboplatin + paclitaxel |
| | M2057 | MVAC |
| | | |
| Both FGFR3 kinase domain mutations and PI3K/mTOR parthway mutations | MR410 | MVAC Durvalumab + Tremelimumab |
| | MR86 | Cisplatin/Carboplatin + gemcitabine |
| | M322 | Carboplatin + gemcitabine MVAC Cisplatin + gemcitabine Paclitaxel |
| | | |
| No FGFR or PI3K/mTOR mutations | MR104 | Cisplatin + gemcitabine MVAC Carboplatin + paclitaxel Gemcitabine |
| | MR103 | Carboplatin + gemcitabine Docetaxel |
| | MR371 | MVAC Atezolizumab |
| | MR411 | Carboplatin + gemcitabine + pembrolizumab |
| | CTC615 | Carboplatin + gemcitabine Pembrolizumab Pazopanib |
Supplementary Table 2. Systemic treatment received by patients prior to selective FGFR inhibitors, according to the detection of FGFR kinase domain mutations and/or PI3K/mTOR pathway alterations.
MVAC: methotrexate, vinblastine, doxorubicin, cisplatin.
