# Supplementary material for: Resistance to selective FGFR inhibitors in FGFR-driven urothelial cancer
Source: Cancer Discov. Author manuscript; Available in PMC 2023 Sep 7. (PMC10481128; doi:10.1158/2159-8290.CD-22-1441)
Supplement: Supplementary table 3 [file EMS178531-supplement-Supplementary_table_3.pptx]

## Slide 1
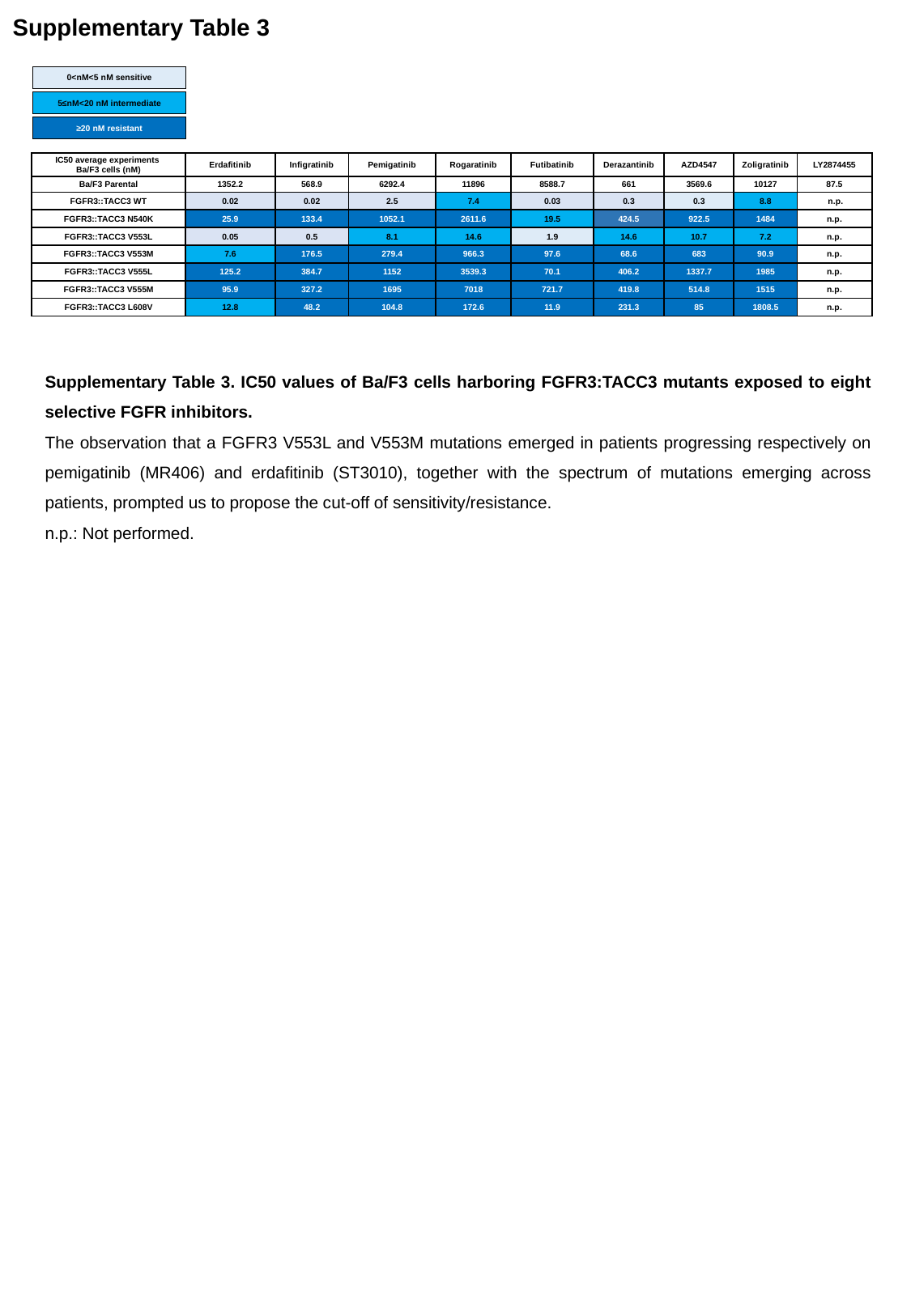

Supplementary Table 3
0<nM<5 nM sensitive
5≤nM<20 nM intermediate
≥20 nM resistant
| IC50 average experiments Ba/F3 cells (nM) | Erdafitinib | Infigratinib | Pemigatinib | Rogaratinib | Futibatinib | Derazantinib | AZD4547 | Zoligratinib | LY2874455 |
| --- | --- | --- | --- | --- | --- | --- | --- | --- | --- |
| Ba/F3 Parental | 1352.2 | 568.9 | 6292.4 | 11896 | 8588.7 | 661 | 3569.6 | 10127 | 87.5 |
| FGFR3::TACC3 WT | 0.02 | 0.02 | 2.5 | 7.4 | 0.03 | 0.3 | 0.3 | 8.8 | n.p. |
| FGFR3::TACC3 N540K | 25.9 | 133.4 | 1052.1 | 2611.6 | 19.5 | 424.5 | 922.5 | 1484 | n.p. |
| FGFR3::TACC3 V553L | 0.05 | 0.5 | 8.1 | 14.6 | 1.9 | 14.6 | 10.7 | 7.2 | n.p. |
| FGFR3::TACC3 V553M | 7.6 | 176.5 | 279.4 | 966.3 | 97.6 | 68.6 | 683 | 90.9 | n.p. |
| FGFR3::TACC3 V555L | 125.2 | 384.7 | 1152 | 3539.3 | 70.1 | 406.2 | 1337.7 | 1985 | n.p. |
| FGFR3::TACC3 V555M | 95.9 | 327.2 | 1695 | 7018 | 721.7 | 419.8 | 514.8 | 1515 | n.p. |
| FGFR3::TACC3 L608V | 12.8 | 48.2 | 104.8 | 172.6 | 11.9 | 231.3 | 85 | 1808.5 | n.p. |
Supplementary Table 3. IC50 values of Ba/F3 cells harboring FGFR3:TACC3 mutants exposed to eight selective FGFR inhibitors.
The observation that a FGFR3 V553L and V553M mutations emerged in patients progressing respectively on pemigatinib (MR406) and erdafitinib (ST3010), together with the spectrum of mutations emerging across patients, prompted us to propose the cut-off of sensitivity/resistance.
n.p.: Not performed.
